# Predicting hospital length of stay using machine learning on a large open health dataset

**DOI:** 10.1186/s12913-024-11238-y

**Published:** 2024-07-29

**Authors:** Raunak Jain, Mrityunjai Singh, A. Ravishankar Rao, Rahul Garg

**Affiliations:** 1 Indian Institute of Technology, Delhi, India; 2 Fairleigh Dickinson University, Teaneck, NJ USA

**Keywords:** Machine learning, Artificial intelligence, Health informatics, Open data, Open-source software, Healthcare analytics

## Abstract

**Background:**

Governments worldwide are facing growing pressure to increase transparency, as citizens demand greater insight into decision-making processes and public spending. An example is the release of open healthcare data to researchers, as healthcare is one of the top economic sectors. Significant information systems development and computational experimentation are required to extract meaning and value from these datasets. We use a large open health dataset provided by the New York State Statewide Planning and Research Cooperative System (SPARCS) containing 2.3 million de-identified patient records. One of the fields in these records is a patient’s length of stay (LoS) in a hospital, which is crucial in estimating healthcare costs and planning hospital capacity for future needs. Hence it would be very beneficial for hospitals to be able to predict the LoS early. The area of machine learning offers a potential solution, which is the focus of the current paper.

**Methods:**

We investigated multiple machine learning techniques including feature engineering, regression, and classification trees to predict the length of stay (LoS) of all the hospital procedures currently available in the dataset. Whereas many researchers focus on LoS prediction for a specific disease, a unique feature of our model is its ability to simultaneously handle 285 diagnosis codes from the Clinical Classification System (CCS). We focused on the interpretability and explainability of input features and the resulting models. We developed separate models for newborns and non-newborns.

**Results:**

The study yields promising results, demonstrating the effectiveness of machine learning in predicting LoS. The best R^2^ scores achieved are noteworthy: 0.82 for newborns using linear regression and 0.43 for non-newborns using catboost regression. Focusing on cardiovascular disease refines the predictive capability, achieving an improved R^2^ score of 0.62. The models not only demonstrate high performance but also provide understandable insights. For instance, birth-weight is employed for predicting LoS in newborns, while diagnostic-related group classification proves valuable for non-newborns.

**Conclusion:**

Our study showcases the practical utility of machine learning models in predicting LoS during patient admittance. The emphasis on interpretability ensures that the models can be easily comprehended and replicated by other researchers. Healthcare stakeholders, including providers, administrators, and patients, stand to benefit significantly. The findings offer valuable insights for cost estimation and capacity planning, contributing to the overall enhancement of healthcare management and delivery.

**Supplementary Information:**

The online version contains supplementary material available at 10.1186/s12913-024-11238-y.

## Introduction

Democratic governments worldwide are placing an increasing importance on transparency, as this leads to better governance, market efficiency, improvement, and acceptance of government policies. This is highlighted by reports from the Organization for Economic Co-operation and Development (OECD) an international organization whose mission it is to shape policies that foster prosperity, equality, opportunity and well-being for all [1]. Openness and transparency have been recognized as pillars for democracy, and also for fostering sustainable development goals [2], which is a major focus of the United Nations (https://sustainabledevelopment.un.org/sdg16).

An important government function is to provide for the healthcare needs of its citizens. The U.S. spends about $3.6 trillion a year on healthcare, which represents 18% of its GDP [3]. Other developed nations spend around 10% of their GDP on healthcare. The percentage of GDP spent on healthcare is rising as populations age. Consequently, research on healthcare expenditure and patient outcomes is crucial to maintain viable national economies. It is advantageous for nations to combine investigations by the private sector, government sector, non-profit agencies, and universities to find the best solutions. A promising path is to make health data open, which allows investigators from all sectors to participate and contribute their expertise. Though there are obvious patient privacy concerns, open health data has been made available by organizations such as New York State Statewide Planning and Research Cooperative System (SPARCS) [4].

Once the data is made available, it needs to be suitably processed to extract meaning and insights that will help healthcare providers and patients. We favor the creation and use of an open-source analytics system so that the entire research community can benefit from the effort [5–7]. As a concrete demonstration of the utility of our system and approach, we revealed that there is a growing incidence of mental health issues amongst adolescents in specific counties in New York State [8]. This has resulted in targeted interventions to address these problems in these communities [8]. Knowing where the problems lie allows policymakers and funding agencies to direct resources where needed.

Healthcare in the U.S. is largely provided through private insurance companies and it is difficult for patients to reliably understand what their expected healthcare costs are [9, 10]. It is ironic that consumers can readily find prices of electronics items, books, clothes etc. online, but cannot find information about healthcare as easily. The availability of healthcare information including costs, incidence of diseases, and the expected length of stay for different procedures will allow consumers and patients to make better and more informed choices. For instance, in the U.S., patients can budget pre-tax contributions to health savings accounts, or decide when to opt for an elective surgery based on the expected duration of that procedure.

To achieve this capability, it is essential to have the underlying data and models that interpret the data. Our goal in this paper is twofold: (a) to demonstrate how to design an analytics system that works with open health data and (b) to apply it to a problem of interest to both healthcare providers and patients. Significant advances have been made recently in the fields of data mining, machine-learning and artificial intelligence, with growing applications in healthcare [11]. To make our work concrete, we use our machine-learning system to predict the length of stay (LoS) in hospitals given the patient information in the open healthcare data released by New York State SPARCS [4].

The LoS is an important variable in determining healthcare costs, as costs directly increase for longer stays. The analysis by Jones [12] shows that the trends in LoS, hospital bed capacity and population growth have to be carefully analyzed for capacity planning and to ensure that adequate healthcare can be provided in the future. With certain health conditions such as cardiovascular disease, the hospital LoS is expected to increase due to the aging of the population in many countries worldwide [13]. During the COVID-19 pandemic, hospital bed capacity became a critical issue [14], and many regions in the world experienced a shortage of healthcare resources. Hence it is desirable to have models that can predict the LoS for a variety of diseases from available patient data.

The LoS is usually unknown at the time a patient is admitted. Hence, the objective of our research is to investigate whether we can predict the patient LoS from variables collected at the time of admission. By building a predictive model through machine learning techniques, we demonstrate that it is possible to predict the LoS from data that includes the Clinical Classifications Software (CCS) diagnosis code, severity of illness, and the need for surgery. We investigate several analytics techniques including feature selection, feature encoding, feature engineering, model selection, and model training in order to thoroughly explore the choices that affect eventual model performance. By using a linear regression model, we obtain an R^2^ value of 0.42 when we predict the LoS from a set of 23 patient features. The success of our model will be beneficial to healthcare providers and policymakers for capacity planning purposes and to understand how to control healthcare costs. Patients and consumers can also use our model to estimate the LoS for procedures they are undergoing or for planning elective surgeries.

## Background

Stone et al. [15] present a survey of techniques used to predict the LoS, which include statistical and arithmetic methods, intelligent data mining approaches and operations-research based methods. Lequertier et al. [16] surveyed methods for LoS prediction.

The main gap in the literature is that most methods focus on analyzing trends in the LoS or predicting the LoS only for specific conditions or restrict their analysis to data from specific hospitals. For instance, Sridhar et al. [17] created a model to predict the LoS for joint replacements in rural hospitals in the state of Montana by using a training set with 127 patients and a test set with 31 patients. In contrast, we have developed our model to predict the LoS for 285 different CCS diagnosis codes, over a set of 2.3 million patients over all hospitals in New York state. The CCS diagnosis code refers to the code used by the Clinical Classifications Software system, which encompasses 285 possible diagnosis and procedure categories [18]. Since the CCS diagnosis codes are too numerous to list, we give a few examples that we analyzed, including but not limited to abdominal hernia, acute myocardial infarction, acute renal failure, behavioral disorders, bladder cancer, Hodgkins disease, multiple sclerosis, multiple myeloma, schizophrenia, septicemia, and varicose veins. To the best of our knowledge, we are not aware of models that predict the LoS on such a variety of diagnosis codes, with a patient sample greater than 2 million records, and with freely available open data. Hence, our investigation is unique from this point of view.

Sotodeh et al. [19] developed a Markov model to predict the LoS in intensive care unit patients. Ma et al. [20] used decision tree methods to predict LoS in 11,206 patients with respiratory disease.

Burn et. al. examined trends in the LoS for patients undergoing hip-replacement and knee-replacement in the U.K. [21]. Their study demonstrated a steady decline in the LoS from 1997–2012. The purpose of their study was to determine factors that contributed to this decline, and they identified improved surgical techniques such as fast-track arthroplasty. However, they did not develop any machine-learning models to predict the LoS.

Hachesu et al. examined the LoS for cardiac disease patients [22] and found that blood pressure is an important predictor of LoS. Garcia et al. determined factors influencing the LoS for undergoing treatment for hip fracture [23]. B. Vekaria et al. analyzed the variability of LoS for COVID-19 patients [24]. Arjannikov et al. [25] used positive-unlabeled learning to develop a predictive model for LoS.

Gupta et al. [26] conducted a meta-analysis of previously published papers on the role of nutrition on the LoS of cancer patients, and found that nutrition status is especially important in predicting LoS for gastronintestinal cancer. Similarly, Almashrafi et al. [27] performed a meta-analysis of existing literature on cardiac patients and reviewed factors affecting their LoS. However, they did not develop quantitative models in their work. Kalgotra et al. [28] use recurrent neural networks to build a prediction model for LoS.

Daghistani et al. [13] developed a machine learning model to predict length of stay for cardiac patients. They used a database of 16,414 patient records and predicted the length of stay into three classes, consisting of short LoS (< 3 days), intermediate LoS ( 3–5 days) and long LoS (> 5 days). They used detailed patient information, including blood test results, blood pressure, and patient history including smoking habits. Such detailed information is not available in the much larger SPARCS dataset that we utilized in our study.

Awad et al. [29] provide a comprehensive review of various techniques to predict the LoS. Though simple statistical methods have been used in the past, they make assumptions that the LoS is normally distributed, whereas the LoS has an exponential distribution [29]. Consequently, it is preferable to use techniques that do not make assumptions about the distribution of the data. Candidate techniques include regression, classification and regression trees, random forests, and neural networks. Rather than using statistical parametric techniques that fit parameters to specific statistical distributions, we favor data-driven techniques that apply machine-learning.

In 2020, during the height of the COVID-19 pandemic, the Lancet, a premier medical journal drew widespread rebuke [30–32] for publishing a paper based on questionable data. Many medical journals published expressions of concern [33, 34]. The Lancet itself retracted the questionable paper [35], which is available at [36] with the stamp “retracted” placed on all pages. One possible solution to prevent such incidents from occurring is for top medical journals to require authors to make their data available for verification by the scientific community. Patient privacy concerns can be mitigated by de-identifying the records made available, as is already done by the New York State SPARCS effort [4]. Our methodology and analytics system design will become more relevant in the future, as there is a desire to prevent a repetition of the Lancet debacle. Even before the Lancet incident, there was declining trust amongst the public related to medicine and healthcare policy [37]. This situation continues today, with multiple factors at play, including biased news reporting in mainstream media [38]. A desirable solution is to make these fields more transparent, by releasing data to the public and explaining the various decisions in terms that the public can understand. The research in this paper demonstrates how such a solution can be developed.

### Requirements

We describe the following three requirements of an ideal system for processing open healthcare data

1. Utilize open-source platforms to permit easy replicability and reproducibility.
2. Create interpretable and explainable models.
3. Demonstrate an understanding of how the input features determine the outcomes of interest.

The first requirement captures the need for research to be easily reproduced by peers in the field. There is growing concern that scientific results are becoming hard for researchers to reproduce [39–41]. This undermines the validity of the research and ultimately hurts the fields. Baker termed this the “reproducibility crisis”, and performed an analysis of the top factors that lead to irreproducibility of research [39]. Two of the top factors consist of the unavailability of raw data and code.

The second requirement addresses the need for the machine-learning models to produce explanations of their results. Though deep-learning models are popular today, they have been criticized for functioning as black-boxes, and the precise working of the model is hard to discern. In the field of healthcare, it is more desirable to have models that can be explained easily [42]. Unless healthcare providers understand how a model works, they will be reluctant to apply it in their practice. For instance, Reyes et al. determined that interpretable Artificial Intelligence systems can be better verified, trusted, and adopted in radiology practice [43].

The third requirement shows that it is important for relevant patient features to be captured that can be related to the outcomes of interest, such as LoS, total cost, mortality rate etc. Furthermore, healthcare providers should be able to understand the influence of these features on the performance of the model [44]. This is especially critical when feature engineering methods are used to combine existing features and create new features.

In the subsequent sections, we present our design for a healthcare analytics system that satisfies these requirements. We apply this methodology to the specific problem of predicting the LoS.

## Methods

**Fig. 1:**
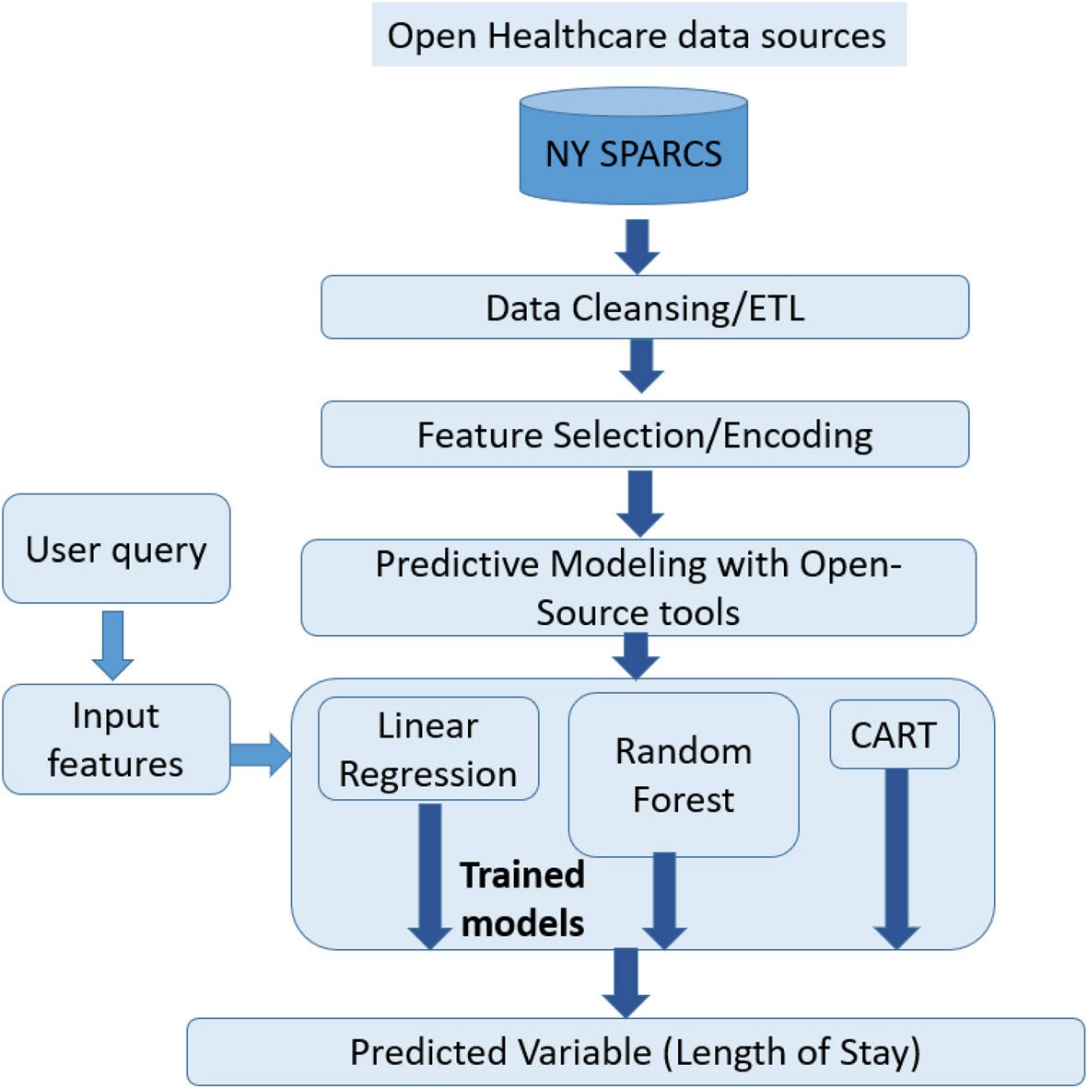
Shows the system architecture. We use Python-based open-source tools such as Pandas and Scikit-Learn to implement the system

We have designed the overall system architecture as shown in Fig. 1. This system is built to handle any open data source. We have shown the New York SPARCS as one of the data sources for the sake of specificity. Our framework can be applied to data from multiple sources such as the Center for Medicare and Medicaid Services (CMS in the U.S.) as shown in our previous work [6]. We chose a Python-based framework that utilizes Pandas [45] and Scikit learn [46]. Python is currently the most popular programming language for engineering and system design applications [47].

**Fig. 2:**
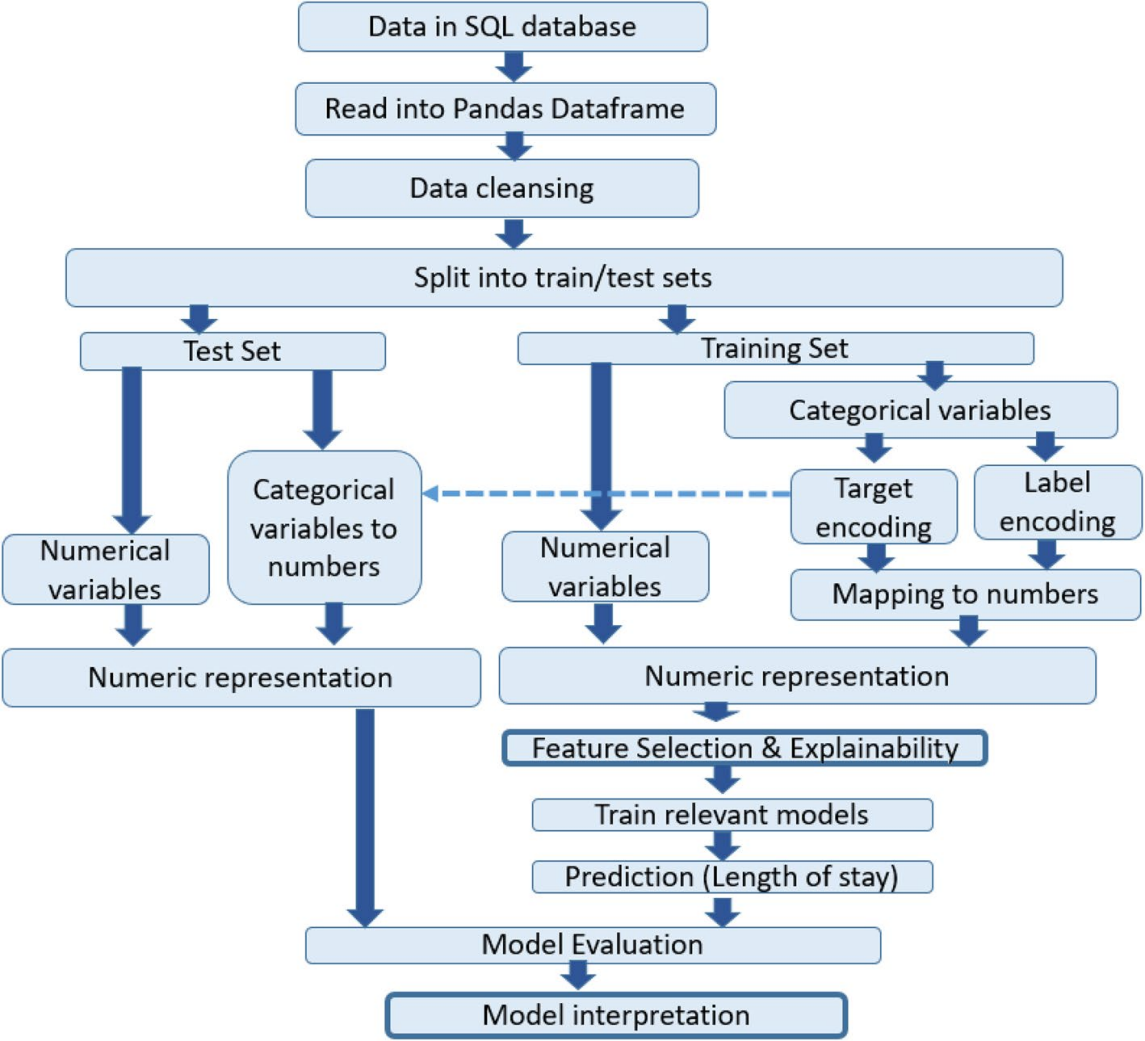
Shows the processing stages in our analytics pipeline

In Fig. 2, we provide a detailed overview of the necessary processing stages. The specific algorithms used in each stage are described in the following sections.

Recent research has shown that it is highly desirable for machine learning models used in the healthcare domain to be explainable to healthcare providers and professionals [48]. Hence, we focused on the interpretability and explainability of input features in our dataset and the models we chose to explore. We restricted our investigation to models that are explainable, including regression models, multinomial logistic regression, random forests, and decision trees. We also developed separate models for newborns and non-newborns.

### Brief description of the dataset

During our investigation, we utilized open-health data provided by the New York State SPARCS system. The data we accessed was from the year 2016, which was the most recent year available at the time. This data was provided in the form of a CSV file, containing 2,343,429 rows and 34 columns. Each row contains de-identified in-patient discharge information. The dataset columns contained various types of information. They included geographic descriptors related to the hospital where care was provided, demographic descriptors such as patient race, ethnicity, and age, medical descriptors such as the CCS diagnosis code, APR DRG code, severity of illness, and length of stay. Additionally, payment descriptors were present, which included information about the type of insurance, total charges, and total cost of the procedure.

Detailed descriptions of all the elements in the data can be found in [49]. The CCS diagnosis code has been described earlier. The term “DRG” stands for Diagnostic Related Group [49], which is used by the Center for Medicare and Medicaid services in the U.S. for reimbursement purposes [50].

The data includes all patients who underwent inpatient procedures at all New York State Hospitals [51]. The payment for the care can come from multiple sources: Department of Corrections, Federal/State/Local/Veterans Administration, Managed Care, Medicare, Medicaid, Miscellaneous, Private Health Insurance, and Self-Pay. The dataset sourced from the New York State SPARCS system, encompassing a wider patient population beyond Medicare/Medicaid, holds greater value compared to datasets exclusively composed of Medicare/Medicaid patients. For instance, Gilmore et al. analyzed only Medicare patients [52].

**Fig. 3:**
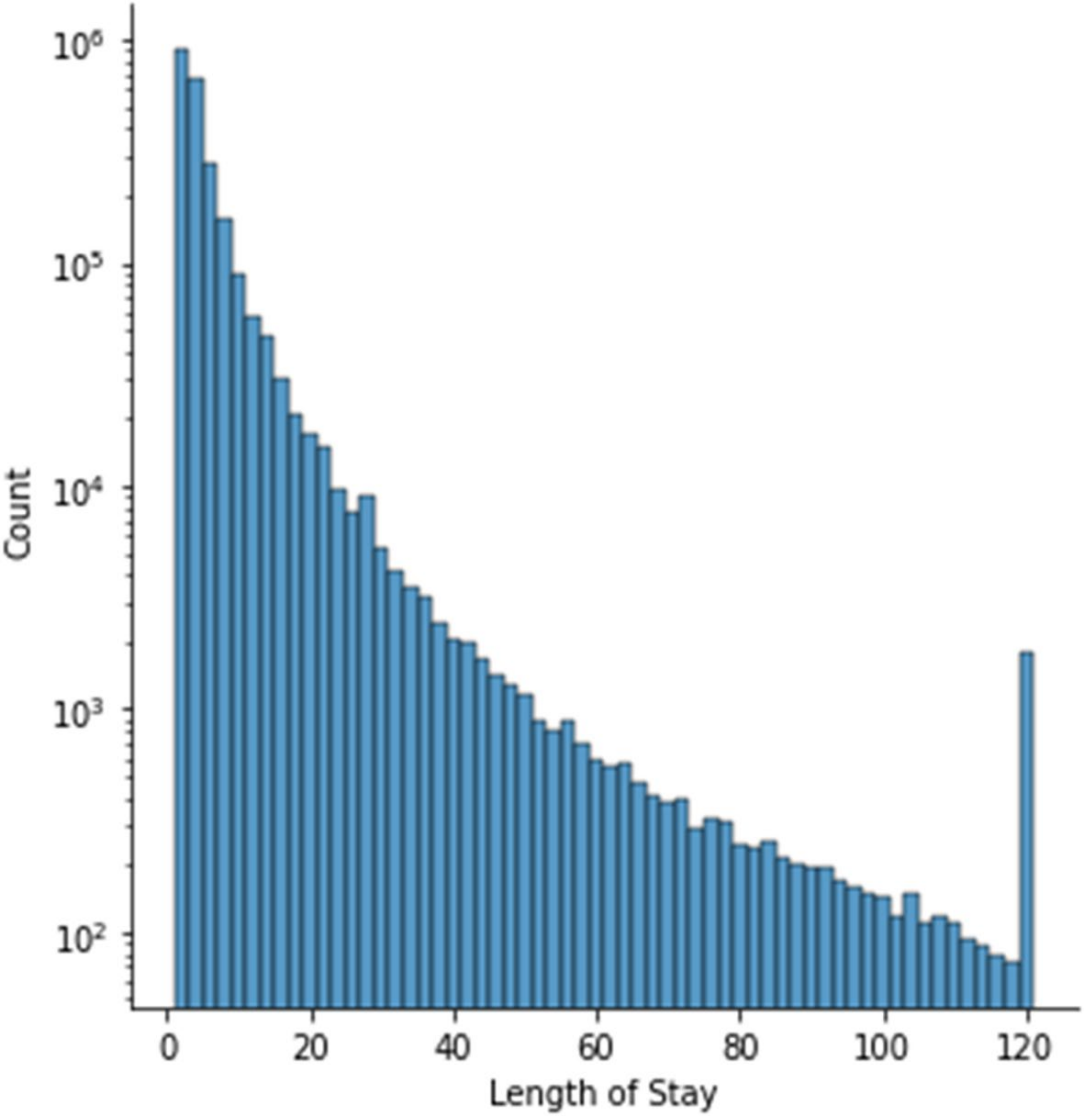
Distribution of the length of stay in the dataset

We examine the distribution of the LoS in the dataset, as shown in Fig. 3. We note that the providers of the data have truncated the length of stay to 120 days. This explains the peak we see at the tail of the distribution.

### Data pre-processing and cleaning

We identified 36,280 samples, comprising 1.55% of the data where there were missing values. These were discarded for further analysis. We removed samples which have Type of Admission = ‘Unknown’ (0.02% samples). So, the final data set has 2,306,668 samples. ‘Payment Typology 2’, and ‘Payment Typology 3’, have missing values (> = 50% samples), which were replaced by a ‘None’ string.

We note that approximately 10% of the dataset consists of rows representing newborns. We treat this group as a separate category. We found that the ‘Birth Weight’ feature had a zero value for non-newborn samples. Accordingly, to better use the ‘Birth Weight’ feature, we partitioned the data into two classes: newborns and non-newborns. This results in two classes of models, one for newborns and the second for all other patients. We removed the ‘Birth Weight’ feature in the input for the non-newborn samples as its value was zero for those samples.

The column ‘Total Costs’ (and in a similar way, ‘Total Charges’) are usually proportional to the LoS, and it would not be fair to use these variables to predict the LoS. Hence, we removed this column. We found that the columns 'Discharge Year', 'Abortion Edit Indicator'' are redundant for LoS prediction models, and we removed them. We also removed the columns ‘CCS Diagnosis Description’, ‘CCS Procedure Description’, ‘APR DRG Description’, ‘APR MDC Description’, and ‘APR Severity of Illness Description’ as we were given their corresponding numerical codes as features.

Since the focus of this paper is on the prediction of the LoS, we analyzed the distribution of LoS values in the dataset.

We developed regression models using all the LoS values, from 1–120. We also developed classification models where we discretized the LoS into specific bins. Since the distribution of LoS values is not uniform, and is heavily clustered around smaller values, we discretized the LoS into a small number of bins, e.g. 6 to 8 bins.

We utilized 10% of the data as a holdout test-set, which was not seen during the training phase. For the remaining 90% of the data, we used tenfold cross-validation in order to train the model and determine the best parameters to use.

### Feature encoding

Many variables in the dataset are categorical, e.g., the variable “APR Severity of Illness Description” has the values in the set [Major, Minor, Moderate, Extreme]. We used distribution-dependent target encoding techniques and one-hot techniques to improve the model performance [53]. We replaced categorical data with the product of mean LoS and median LoS for a category value. The categorical feature can then better capture the dependence distribution of LoS with the value of the categorical feature.

For the linear regression model [54], we sampled a set of 6 categorical features, [‘Type of Admission’, ‘Patient Disposition’, ‘APR Severity of Illness Code’, ‘APR Medical Surgical Description’, ‘APR MDC Code’] which we target encoded with the mean of the LoS and the median of the LoS. We then one-hot encoded every feature (all features are categorical) and for each such one-hot encoded feature, created a new feature for each of the features in the sampled set, by replacing the ones in the one-hot encoded feature with the value of the corresponding feature in the sampled set. For example, we one-hot encoded ‘Operating Certificate Number’, and for samples where ‘Operating Certificate Number’ was 3, we created 6 features, each where samples having the value 3 were assigned the target encoded values of the sampled set features, and the other samples were assigned zero. We used such techniques to exploit the linear relation between LoS and each feature.

According to the sklearn documentation [55], a random forest regressor is “a meta estimator that fits a number of decision tree regressors on various sub-samples of the dataset and uses averaging to improve the predictive accuracy and control over-fitting”. The random forest regressor leverages ensemble learning based on many randomized decision trees to make accurate and robust predictions for regression problems. The averaging of many trees protects against single trees overfitting the training data.

The random forest classifier is also an ensemble learning technique and uses many randomized decision trees to make predictions for classification problems. The 'wisdom of crowds' concept suggests that the decision made by a larger group of people is typically better than an individual. The random forest classifier uses this intuition, and allows each decision tree to make a prediction. Finally, the most popular predicted class is chosen as the overall classification.

For the Random Forest Regressor [56, 57] and Random Forest Classifier [58], we only used a similar distribution dependent target encoding as a random forest classifier/ regressor is unsuitable for sparse one-hot encoded columns.

Multinomial logistic regression is a type of regression analysis that predicts the probabilities of the different possible outcomes of a categorically distributed dependent variable, given a set of independent variables. It allows for more than two discrete outcomes, extending binomial logistic regression for binary classification to models with multiple class membership. For the multinomial logistic regression model [59], we used only one-hot encoding, and not target encoding, as the target value was categorical.

Finally, we experimented with combinations of target encoding and one-hot encoding. We can either use target encoding, or one-hot encoding, or both. When both encodings are employed, the dimensionality of the data increases to accommodate the one-hot encoded features. For each combination of encodings, we also experimented with different regression models including linear regression and random forest regression.

### Feature importance, selection, and feature engineering

We experimented with different feature selection methods. Since the focus of our work is on developing interpretable and explainable models, we used SHAP analysis to determine relevant features.

We examine the importance of different features in the dataset. We used the SHAP value (Shapley Additive Explanations), a popular measure for feature importance [60]. Intuitively, the SHAP value measures the difference in model predictions when a feature is used versus omitted. It is captured by the following formula.

$$\emptyset_i(p)=\sum\limits_{S\subseteq N/i}\frac{|S|!(n-|S|-1)!}{n!}(p(S\cup i)-p(S))$$

where ${{\varnothing }}_{i}$ is the SHAP value of feature $i$, $p$ is the prediction by the model, n is the number of features and S is any set of features that does not include the feature $i$. The specific model we used for the prediction was the random forest regressor where we target-encoded all features with the product of the mean and the median of the LoS, since most of the features were categorical.

### Classification models

**Fig. 4:**
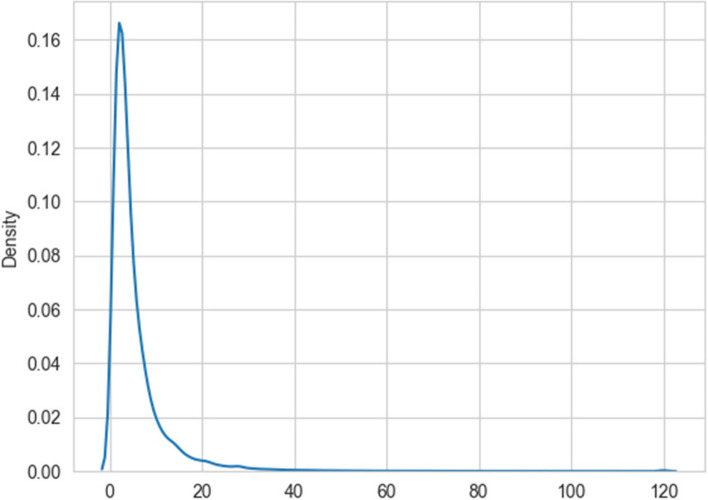
A density plot of the distribution of the length of stay. The area under the curve is 1. We used a kernel density estimation with a Gaussian kernel [61] to generate the plot

One approach to the problem is to bin the LoS into different classes, and train a classifier to predict which class an input sample falls in. We binned the LoS into roughly balanced classes as follows: 1 day, 2 days, 3 days, 4–6 days, > 6 days. This strategy is based on the distribution of the LoS as shown earlier in Figs. 3 and 4.

We used three different classification models, comprising the following:

1. Multinomial Logistic Regression
2. Random Forest Classifier
3. CatBoost classifier [62].

We used a Multinomial Logistic Regression model [59] trained and tested using tenfold cross validation to classify the LoS into one of the bins. The multinomial logistic regression model is capable of providing explainable results, which is part of the requirements. We used the feature engineering techniques described in the previous section.

We used a Random Forest Classifier model trained and tested using tenfold cross validation to classify the LoS into one of the bins. We used a maximum depth of 10 so as to get explainable insights into the model.

Finally, we used a CatBoost Classifier model trained and tested using tenfold cross validation to classify the LoS into one of the bins.

### Regression models

We used three different regression models with the feature engineering techniques mentioned above (Feature encoding section). These comprise:

1. Linear regression
2. Catboost regression
3. Random forest regression

The linear regression was implemented using the nn.Linear() function in the open source library PyTorch [63]. We used the ‘Adam’ optimization algorithm [64] in mini-batch settings to train the model weights for linear regression.

We investigated CatBoost regression in order to create models with minimal feature sets, whereby models with a low number of input features would provide adequate results. Accordingly, we trained a CatBoost Regressor [65] in order to determine the relationship between combinations of features and the prediction accuracy as determined by the R^2^ correlation score.

The random forest regression was implemented using the function RandomForestRegressor() in scikit learn [55].

### Model performance measures

For the regression models, we used the following metrics to compare the model performance.

1. The R^2^ score and the *p*-value. We use a significance level of α = 0.05 (5 %) for our statistical tests.  If the *p*-value is small, i.e. less than α = 0.05, then the R^2^ score is statistically significant.

For classifier models, we used the following metrics to compare the model performance.

1. True positive rate, false negative rate, and F1 score [66].
2. We computed the Brier score using Brier’s original calculation in his paper [67]. In this formulation, for R classes the Brier score B can vary between 0 and R, with 0 being the best score possible.
  $$B= \frac{1}{N}{\sum }_{i}{\sum }_{c}{( {\widehat{y}}_{i,c} - {I}_{i,c})}^{2}$$
  where ${\widehat{y}}_{i,c}$ is the class probability as per the model and ${I}_{i,c}=1$ if the *i* th sample belongs to class *c* and ${I}_{i,c}=0$ if it does not belong to class *c*.
3. We used the Delong test [68] to compare the AUC for different classifiers.

These metrics will allow other researchers to replicate our study and provide benchmarks for future improvements.

## Results

In this section we present the results of applying the techniques in the Methods section.

### Descriptive statistics

We provide descriptive statistics that help the reader understand the distributions of the variables of interest.

Table 1 summarizes basic statistical properties of the LoS variable.

**Table 1:** Descriptive statistics regarding the LoS variable

Mean	5.41
std. deviation	7.97
Minimum	1
25th percentile	2
50th percentile	3
75th percentile	6
Maximum	120

**Fig. 5:**
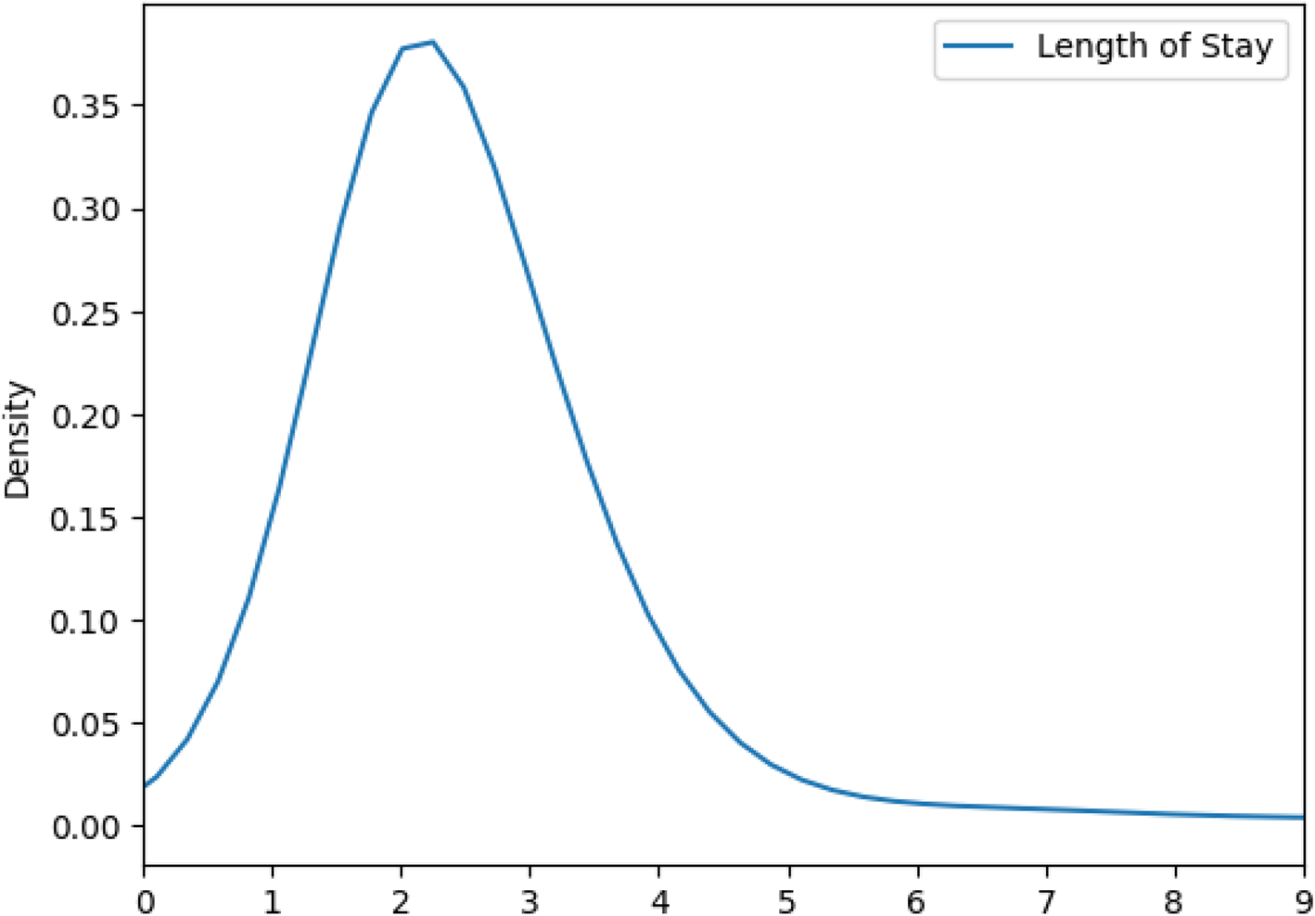
This figure depicts the distribution of the LoS variable for newborns

Figure 5 shows the distribution of the LoS variable for newborns.

Table 2 shows the top 20 APR DRG descriptions based on their frequency of occurrence in the dataset.

**Table 2:** This table depicts the frequency of occurrence of the top 20 APR DRG descriptions in the dataset

APR DRG Description	Frequency
Neonate birthwt > 2499 g, normal newborn or neonate w other problem	195,238
Vaginal delivery	142,275
Septicemia & disseminated infections	93,349
Cesarean delivery	74,561
Heart failure	56,708
Other pneumonia	40,890
Knee joint replacement	39,824
Chronic obstructive pulmonary disease	38,023
Schizophrenia	36,329
Cellulitis & other skin infections	33,235
Hip joint replacement	32,888
Cardiac arrhythmia & conduction disorders	32,472
Kidney & urinary tract infections	29,801
RENAL FAILURE	29,118
CVA & precerebral occlusion w infarct	25,731
Bipolar disorders	25,429
Seizure	25,290
Major depressive disorders & other/unspecified psychoses	23,541
Percutaneous coronary intervention w/o AMI	22,261
Alcohol abuse & dependence	22,151

**Fig. 6:**
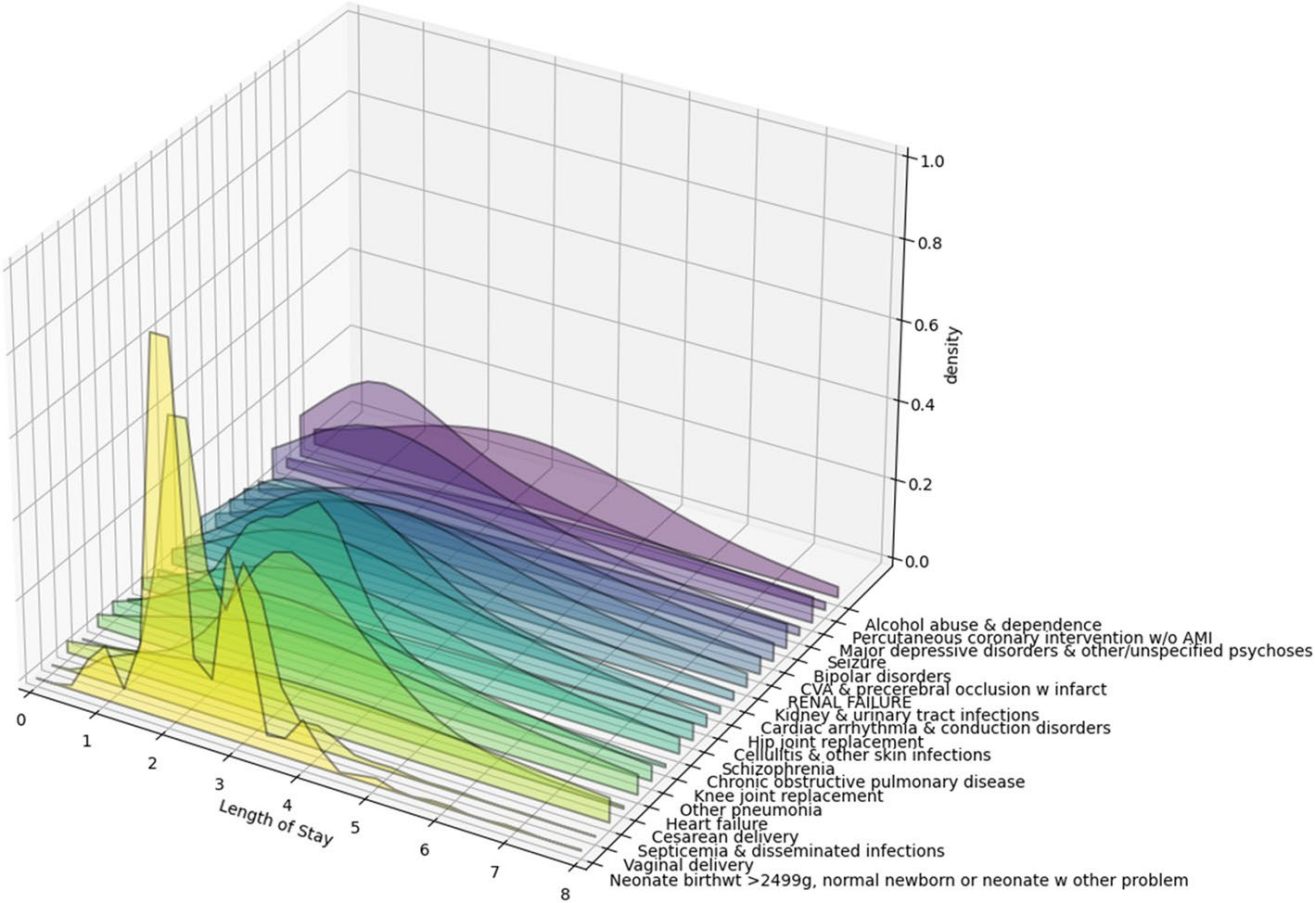
A 3-d plot showing the distribution of the LoS for the top-20 most frequently occuring APR DRG descriptions. The x-axis (horizontal) depicts the LoS, the y-axis shows the APR DRG codes and the z-axis shows the density or frequency of occurrence of the LoS

Figure 6 shows the distribution of the LoS variable for the top 20 most frequently occurring APR DRG descriptions shown in Table 2.

### Feature encoding

We experimented with different encoding schemes for the categorical variables and for each encoding we examined different regression techniques. Our results are shown in Table 3. We experimented with the three encoding schemes shown in the first column. The last row in the table shows a combination of one-hot encoding and target encoding, where the number of columns in the dataset are increased to accommodate one-hot encoded feature values for categorical variables.

**Table 3:** The regression results produced by varying the encoding scheme and the model. This data is for non-newborns

Encodings	Model	R^2^ Score
One Hot	Linear Regression	0.36
Target	Random Forest Regressor	0.396
One Hot and Target	Linear Regression	0.42

### Feature importance, selection and feature engineering

We obtained the SHAP plots using a Random Forest Regressor trained with target-encoded features.

**Fig. 7:**
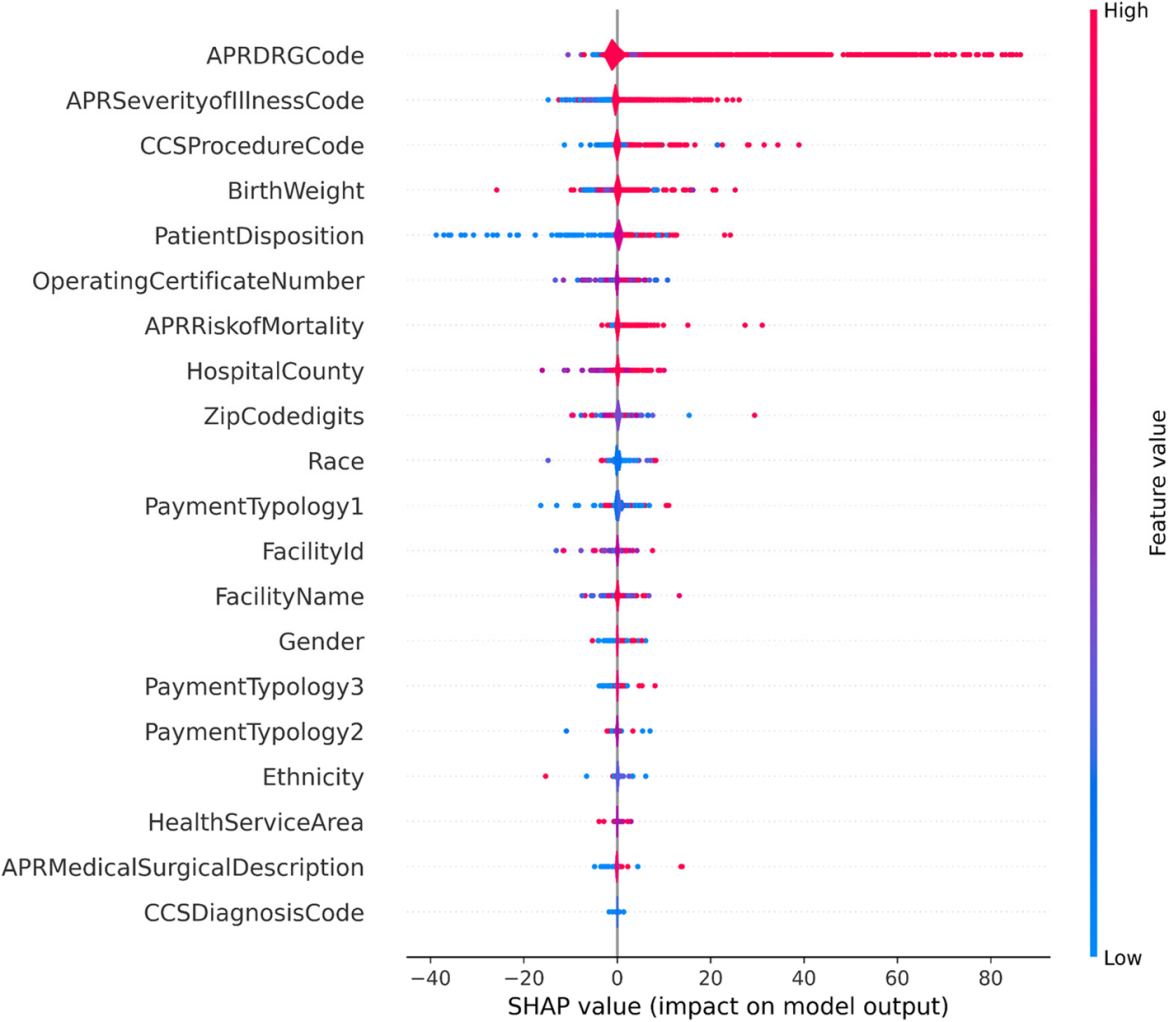
SHAP Value plot for newborns

**Fig. 8:**
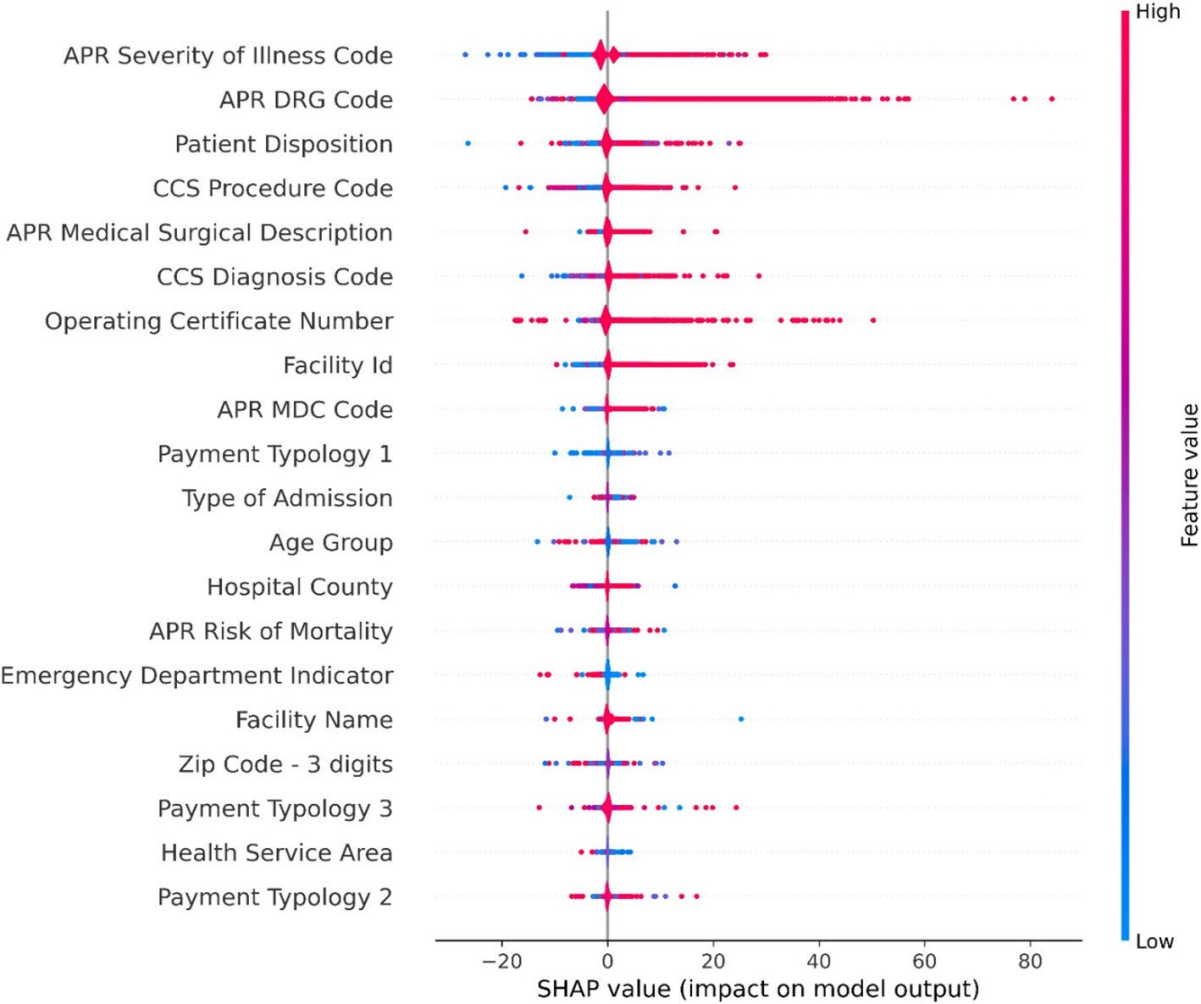
1-D SHAP plot, in order of decreasing feature importance: top to bottom (for non-newborns)

Figures 7 and 8 show the SHAP values plots obtained for the features in the newborn partition of the dataset. We find that the features, “APR DRG Code”, “APR Severity of Illness Code”, “Patient Disposition”, “CCS Procedure Code”, are very useful in predicting the LoS. For instance, high feature values for “APR Severity of Illness Code”, which are encoded by red dots have higher SHAP values than the blue dots, which correspond to low feature values.

A similar interpretation can be applied to the features in the non-newborn partition of the dataset. We note that “Operating Certificate Number” is among the top-10 most important features in both the newborn and non-newborn partitions. This finding is discussed in the Discussion section.

**Fig. 9:**
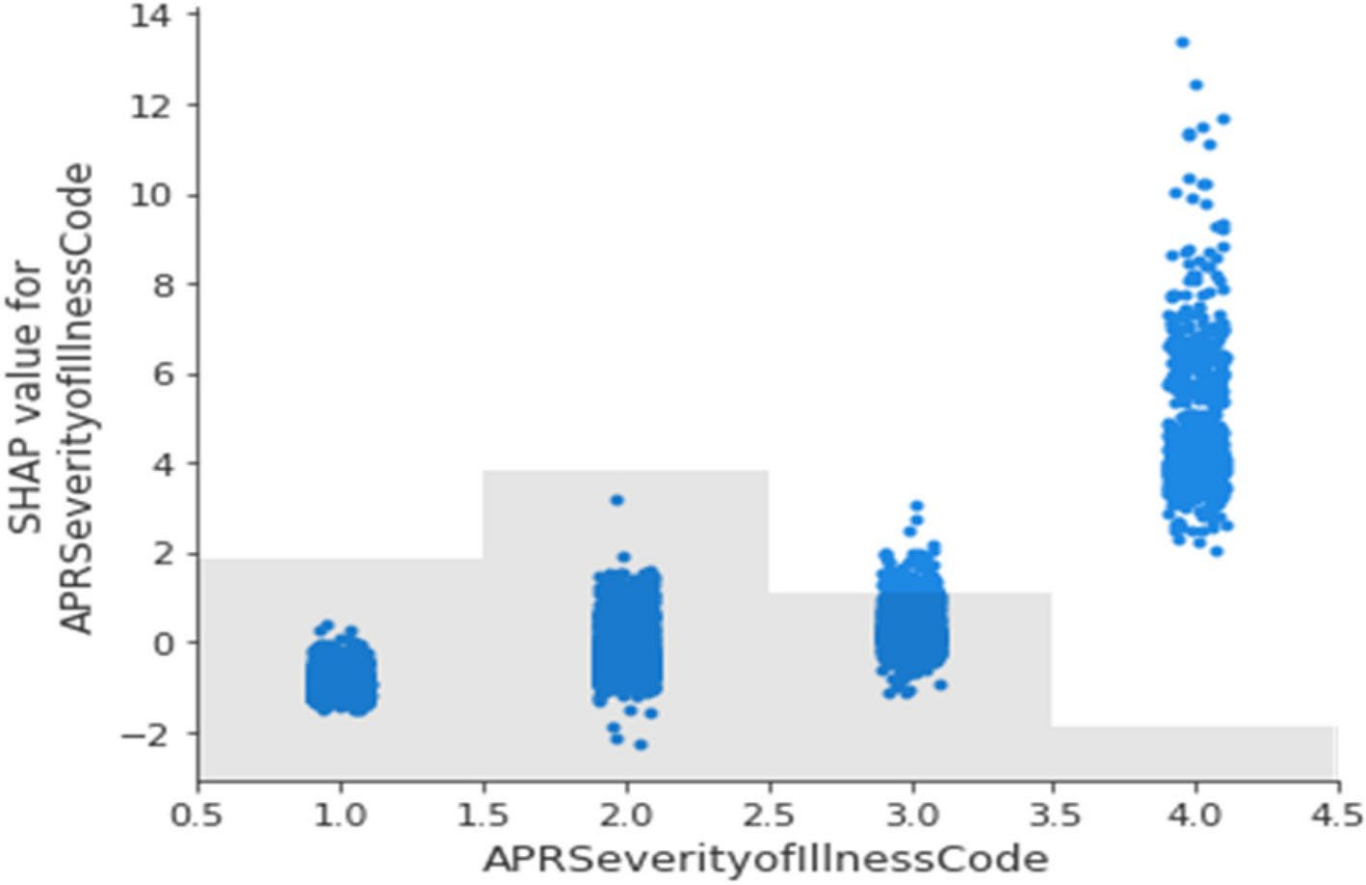
A 2-D plot showing the relationship between SHAP values for one feature, “APR Severity of Illness Code”, and the feature values themselves (non-newborns)

From Fig. 9, we observe that as the severity of illness code increases from 1–4, there is a corresponding increase in the SHAP values.

**Fig. 10:**
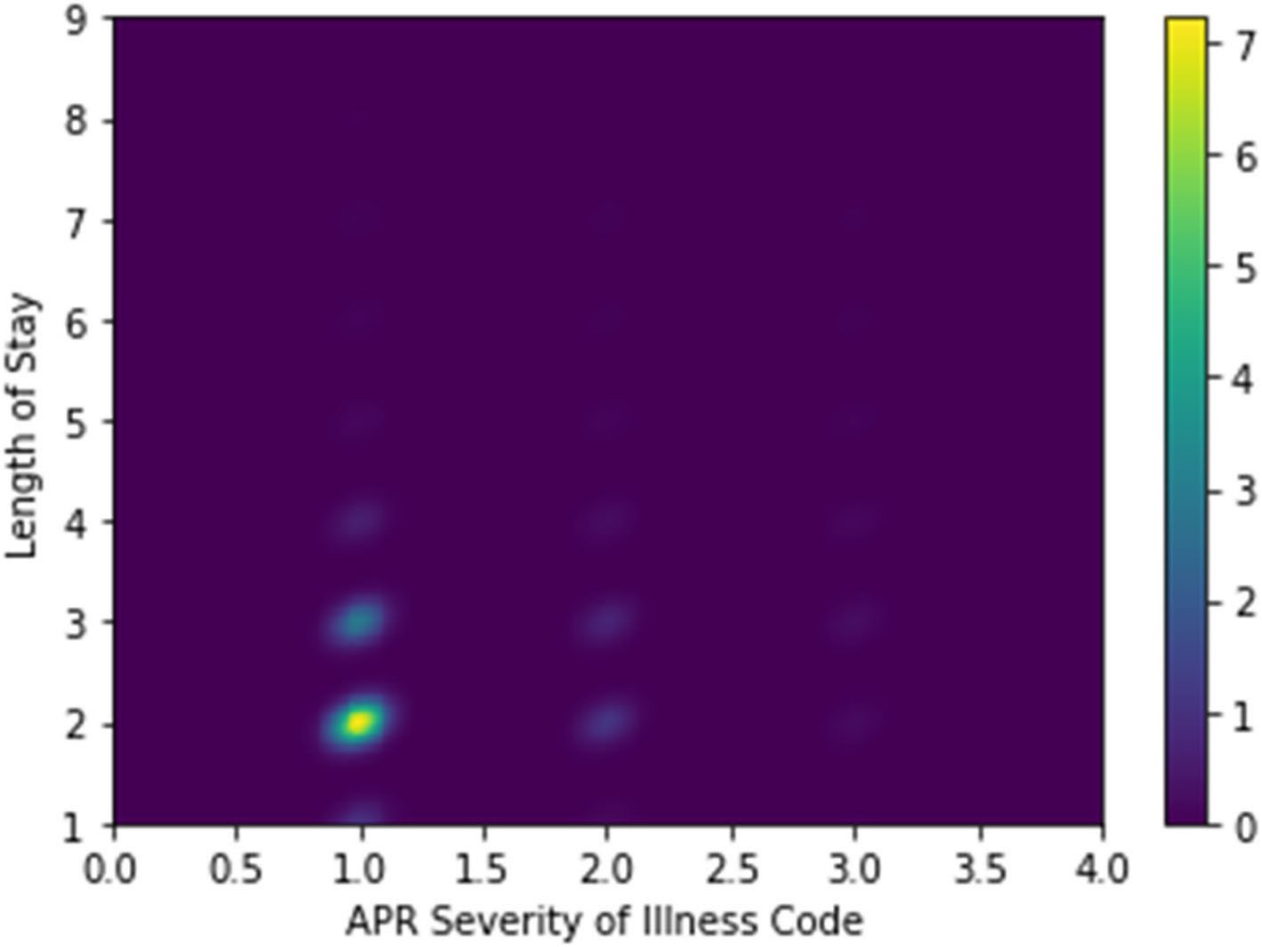
A density plot showing the relationship between APR Severity of Illness Code and the LoS. The color scale on the right determines the interpretation of colors in the plot. We used a kernel density estimation with a Gaussian kernel [61] to generate the plot

To further understand the relationship between the APR Severity of Illness code and the LoS, we created the plot in Fig. 10. This shows that the most frequently occurring APR Severity of Illness code is 1 (Minor), and that the most frequently occurring LoS is 2 days. We provide this 2-D projection of the overall distribution of the multi-dimensional data as a way of understanding the relationship between the input features and the target variable, LoS.

**Fig. 11:**
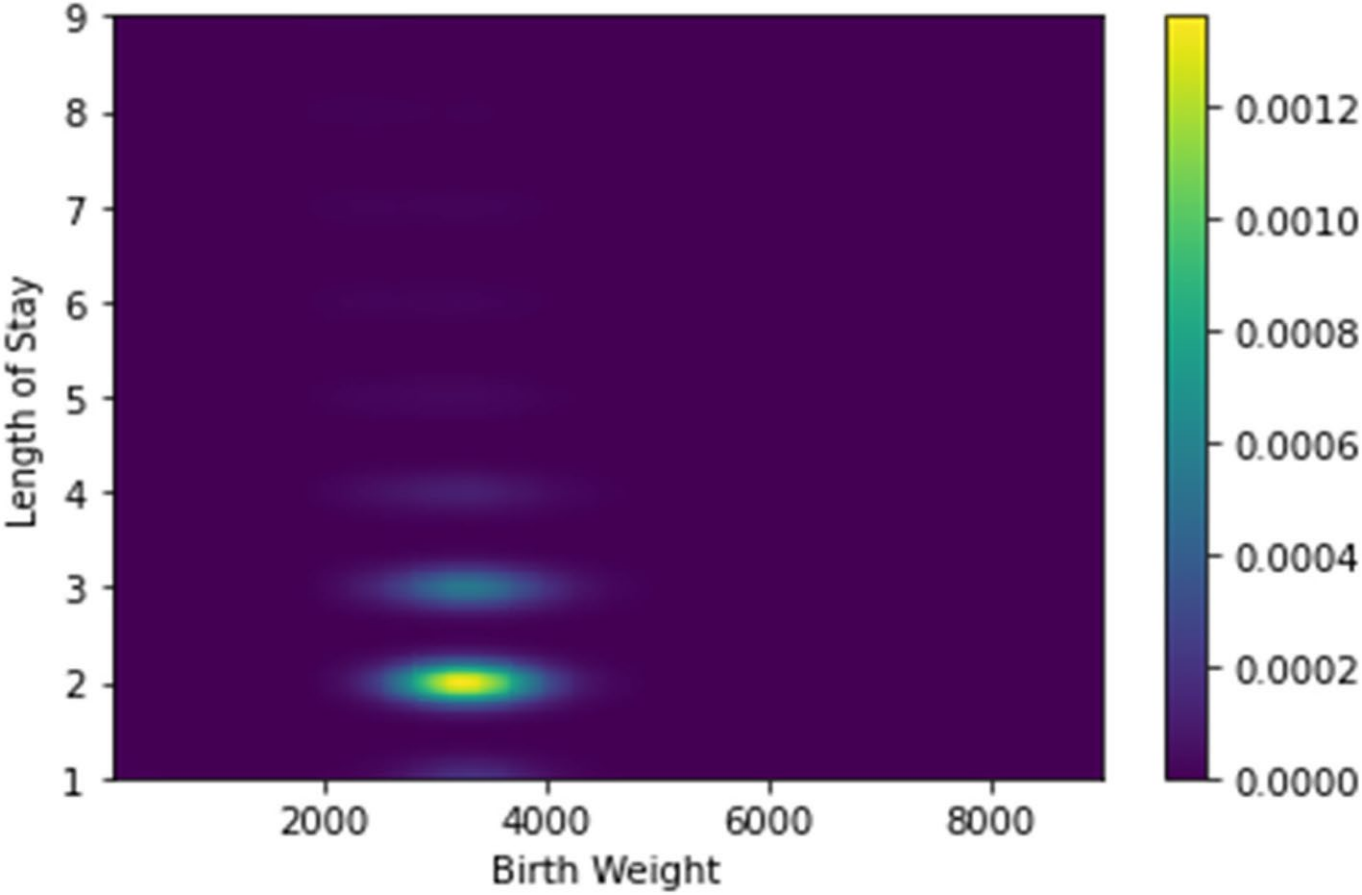
A density plot showing the distribution of the birth weight values (in grams) versus the LoS. The colorbar on the right shows the interpretation of color values shown in the plot. We used a kernel density estimation with a Gaussian kernel [61] to generate the plot

Similarly, Fig. 11 shows the relationship between the birth weight and the length of stay. The most common length of stay is two days.

### Classification

**Fig. 12:**
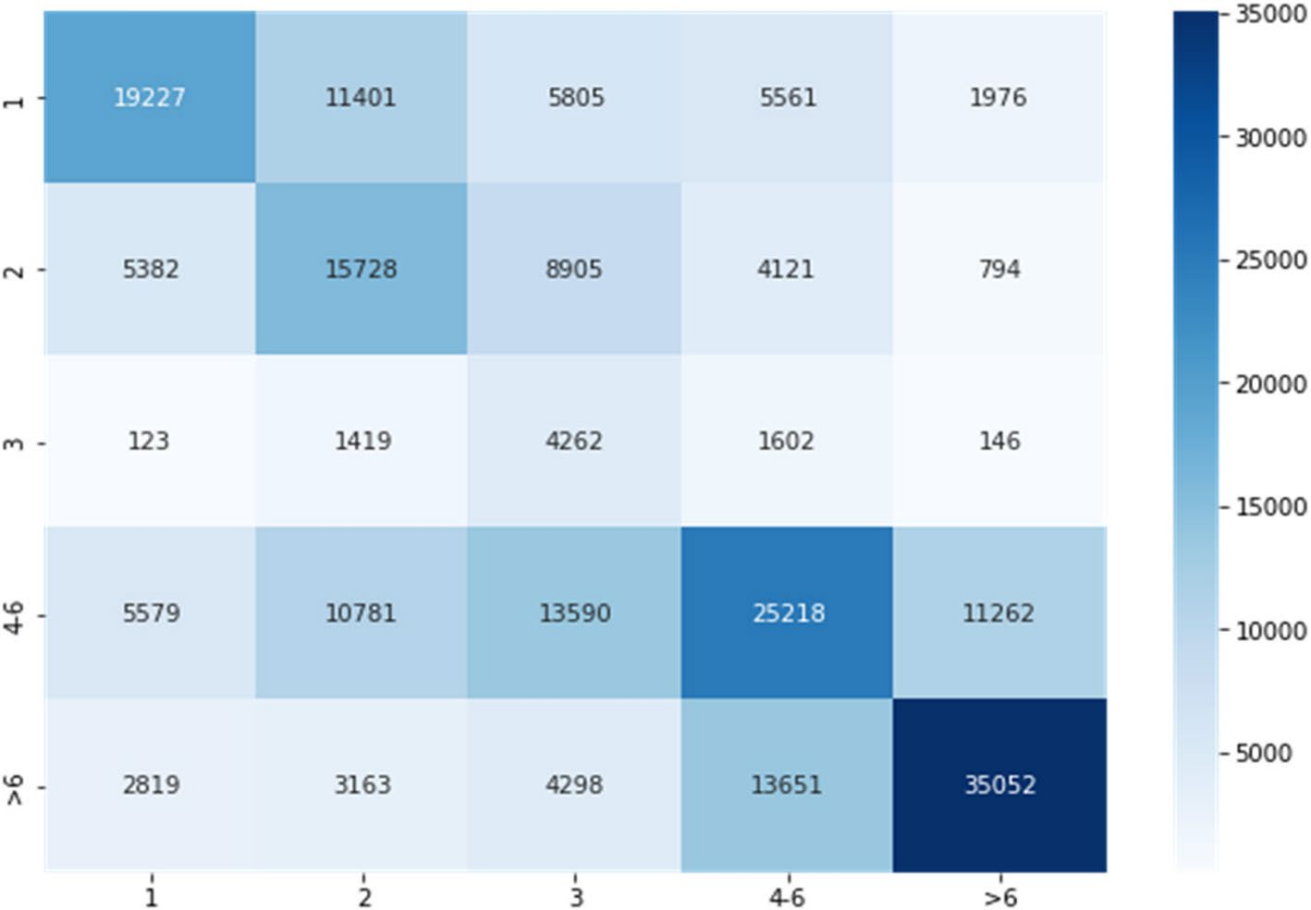
Confusion matrix for classification of non-newborns. The number inside each square along the diagonal represents the number of correctly classified samples. The color is coded so lighter colors represent lower numbers

We obtained a classification accuracy of 46.98% using Multinomial Logistic Regression with tenfold cross-validation in the 5-class classification task for non-newborn cases. The confusion matrix in Fig. 12 shows that the highest density of correctly classified samples is in or close to the diagonal region. The regions where out model fails occurs between adjacent classes as can be inferred from the given confusion matrix.

**Fig. 13:**
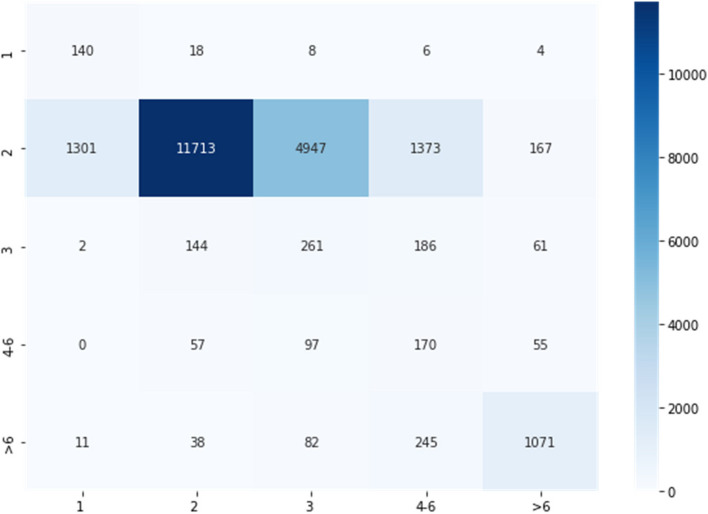
Confusion matrix for classification of newborns. The number inside each square along the diagonal represents the number of correctly classified samples. The color is coded so lighter colors represent lower numbers

**Fig. 14:**
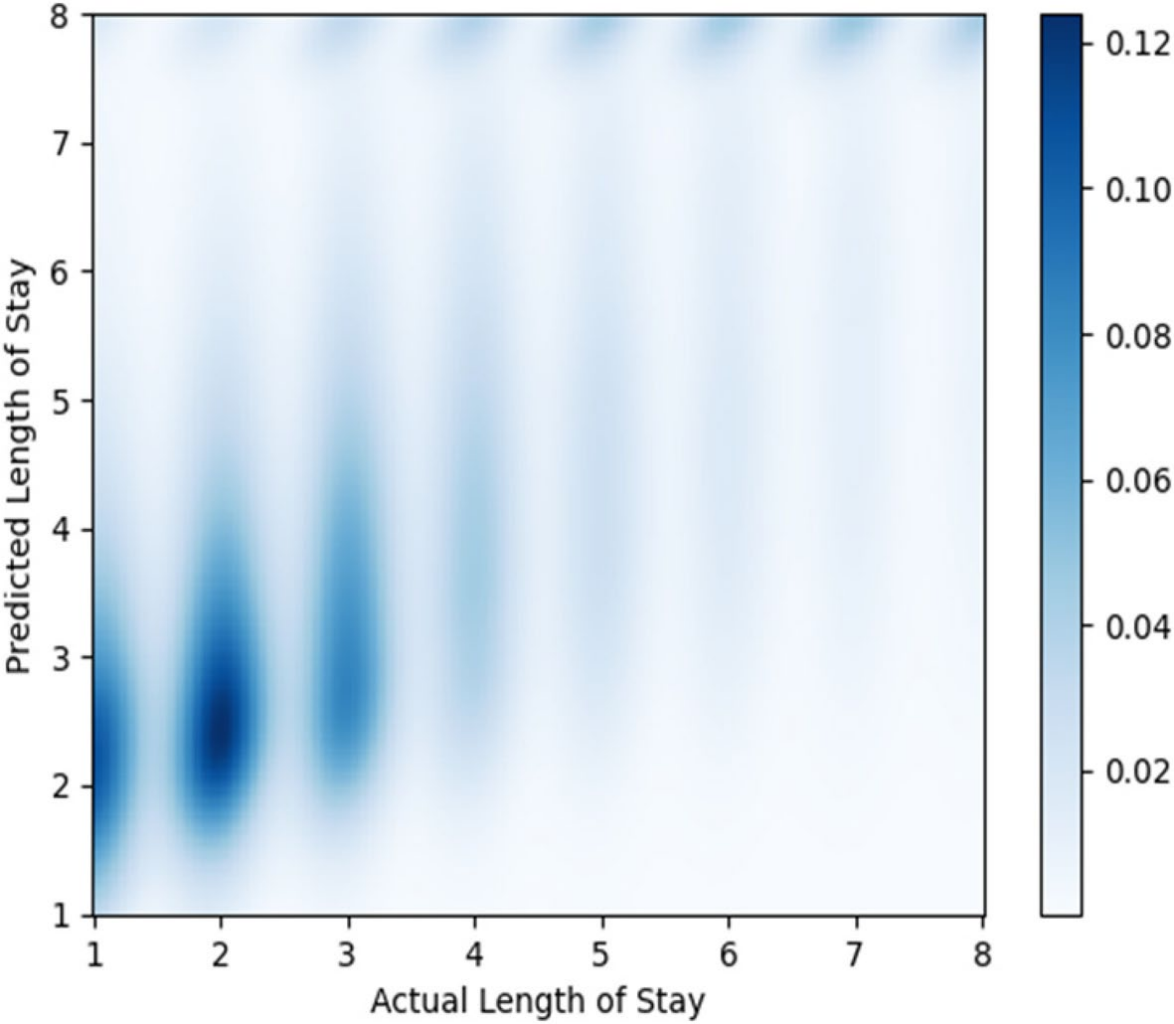
Shows the density plot of the predicted length of stay versus actual length of stay for the classifier model for non-newborns. We used a kernel density estimation with a Gaussian kernel [61] to generate the plot

For the newborn cases, we obtained a classification accuracy of 60.08% using Random Forest Classification model with tenfold cross-validation in the 5-class classification task. The confusion matrix in Fig. 13 shows that the majority of data samples lie in or close to the diagonal region. The regions where our model does not do well occurs between adjacent classes as can be inferred from the given confusion matrix,

The density plot in Fig. 14 shows the relationship between the actual LoS and the predicted LoS. For a LoS of 2 days, the centroid of the predicted LoS cluster is between 2 and 3 days.

**Fig. 15:**
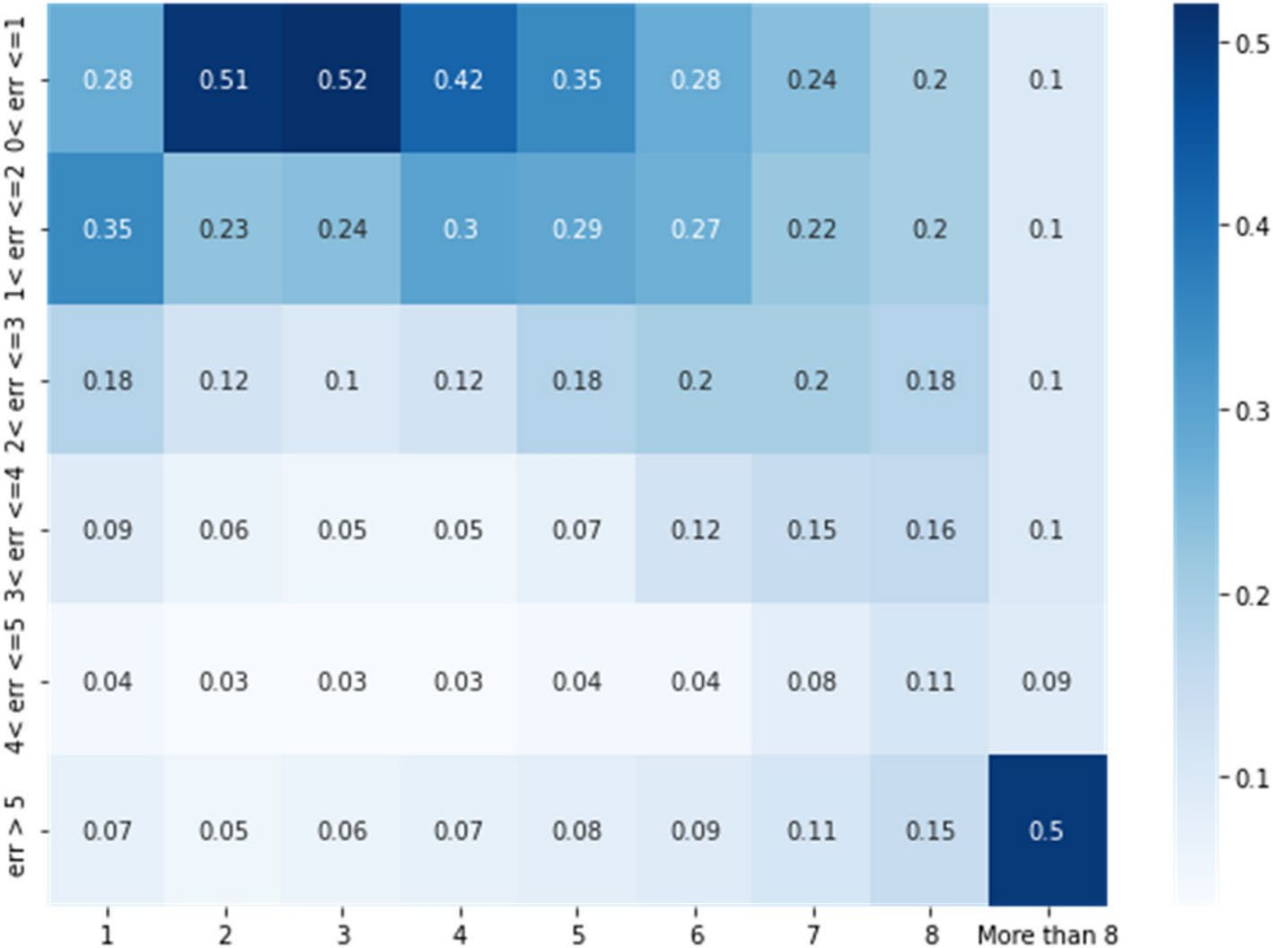
Shows the distribution of correctly predicted LoS values for each class used in our model. Along the columns, we depict the different classes used in the model, consisting of LoS equal to 1, 2, 3 …8, and more than 8. Each row depicts different errors made in the prediction. For instance, the top row depicts an error of less than or equal to one day between the actual LoS and the predicted Los. The second row from the top depicts an error which is greater than 1 and less than or equal 2 days. And so on for the other rows, for non-newborns

A quantitative depiction of our model errors is shown in Fig. 15. The values in Fig. 15 are interpreted as follows. Referring to the column for LoS = 2, the top row shows that 51% of the predicted LoS values for an actual stay of 2 days is also 2 days (zero error), and that 23% of the predicted values for LoS equal to 2 days have an error of 1 day and so on. The relatively high values in the top row indicates that the model is performing well, with an error of less than 1 day. There are relatively few instances of errors between 2 and 3 days (typically less than 10% of the values show up in this row). The only exception is for the class corresponding to LoS great than 8 days. The truncation of the data to produce this class results in larger model errors specifically for this class.

### Regression

**Fig. 16:**
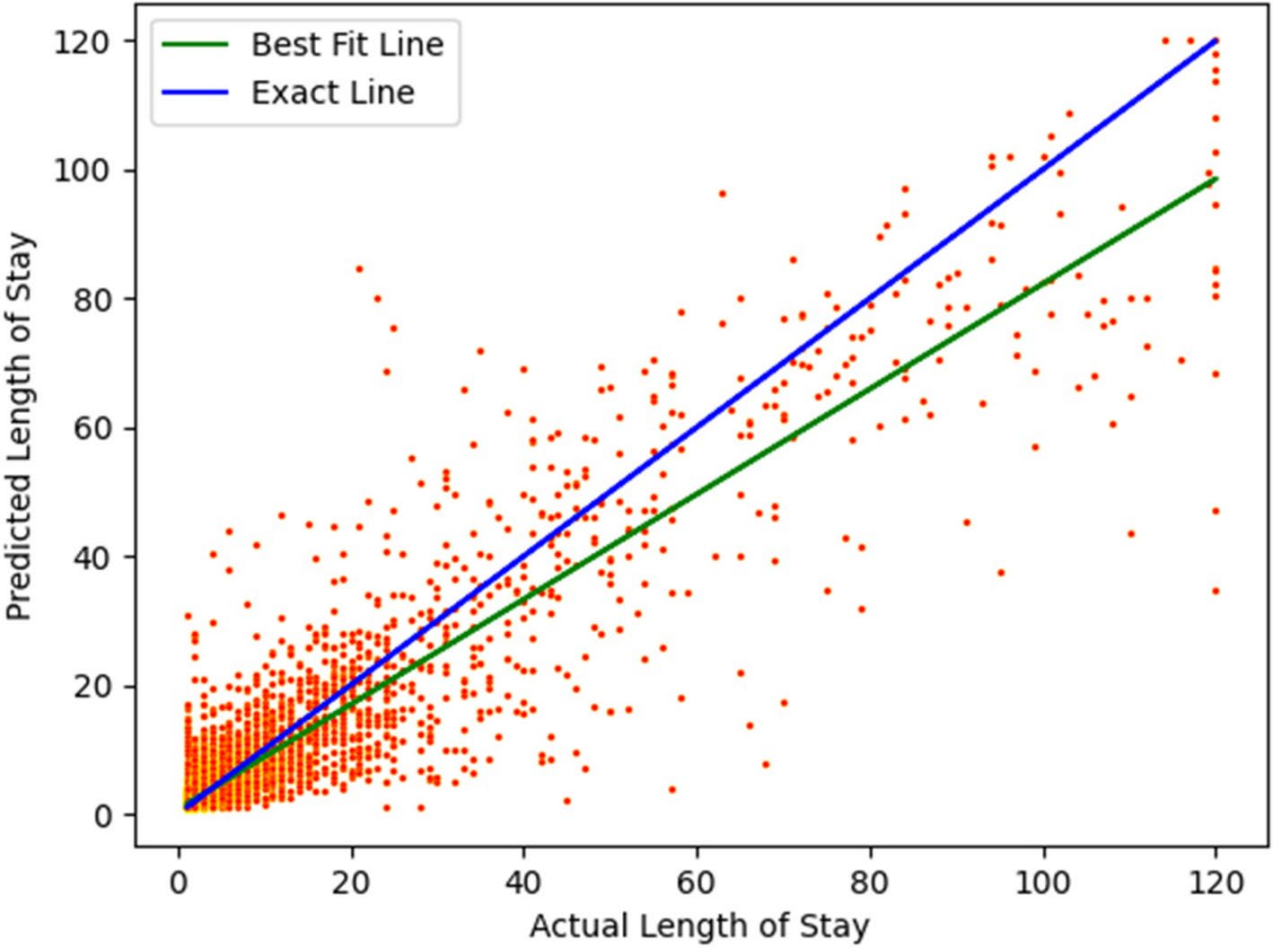
Scatter plot showing an instance of a linear regression fit to the data (newborns). The R^2^ score is 0.82. The blue line represents an exact fit, where the predicted LoS equals the actual LoS (slope of the line is 1)

**Fig. 17:**
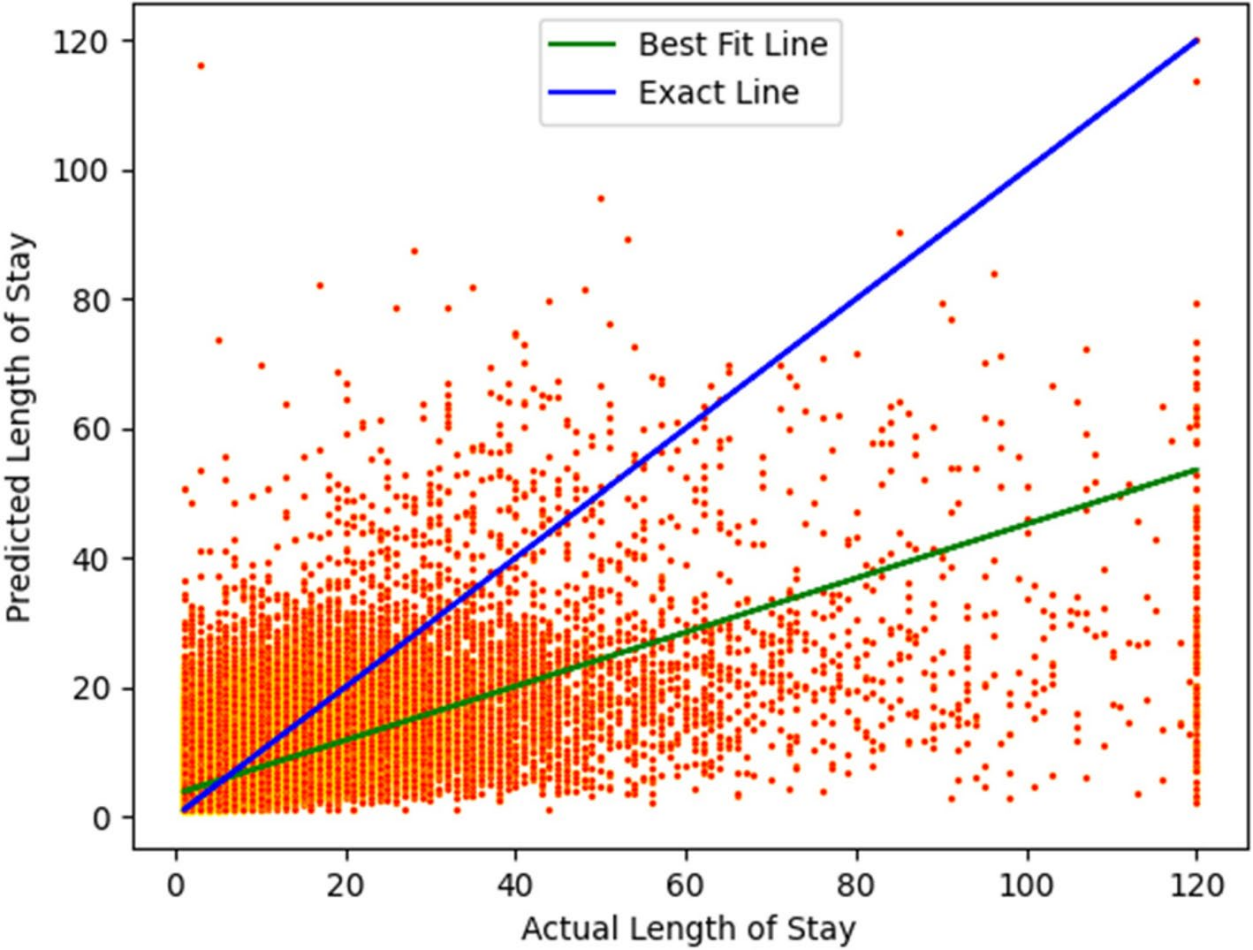
Scatter plot for linear regression. (non-newborns). The R^2^ score is 0.42. The blue line represents an exact fit, where the predicted LoS equals the actual LoS (slope of the line is 1)

Figures 16 and 17 show the scatter plots for the linear regression models. The exact line represents a line with slope 1, and a perfect model would be one that produced all points lying on this line.

**Fig. 18:**
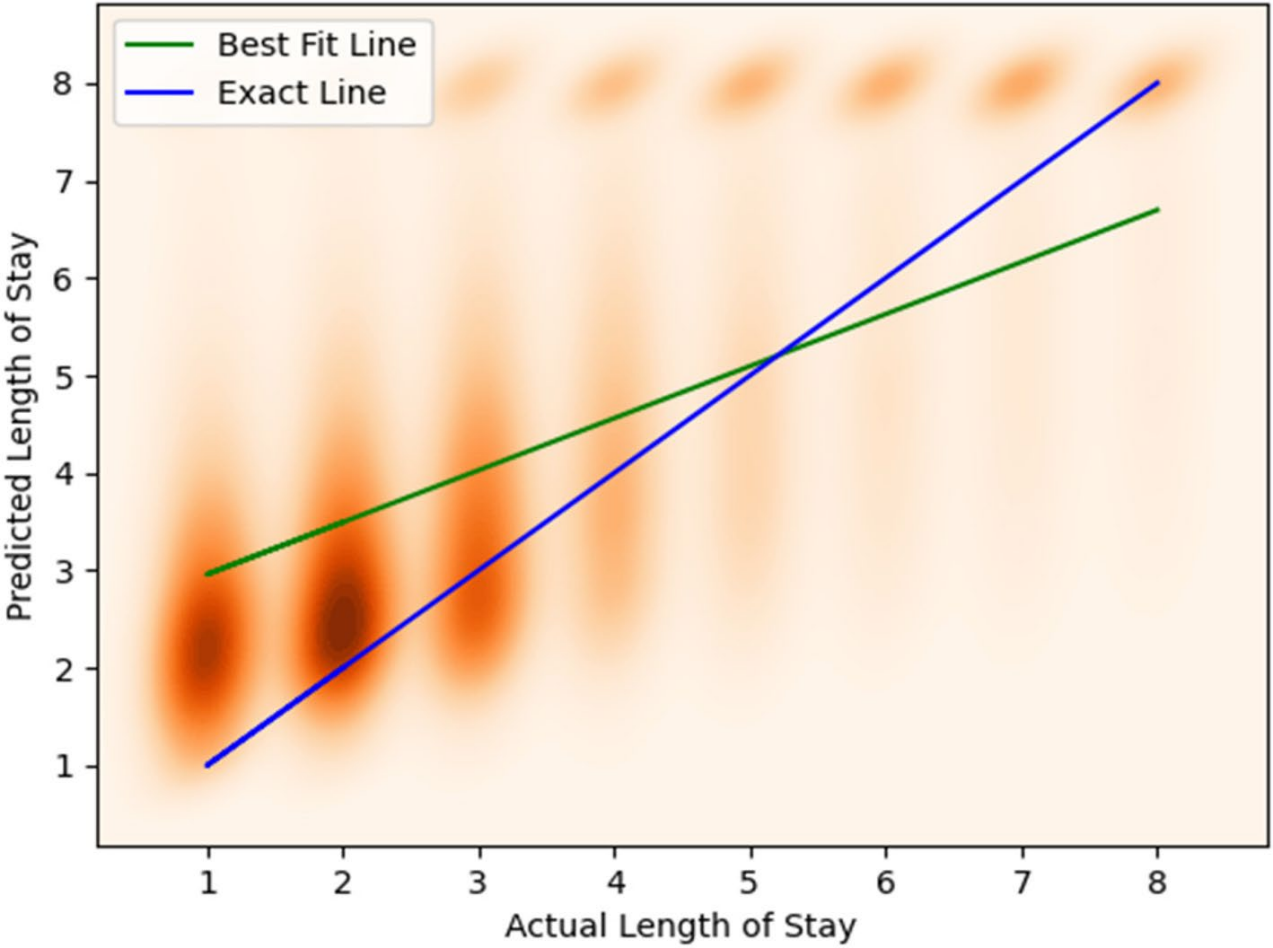
Shows the density plot of the predicted length of stay versus actual length of stay for the classifier model for non-newborns. We used a kernel density estimation with a Gaussian kernel [40] to generate the plot. The best fit regression line to our predictions is shown in green, whereas the blue line represents the ideal fit (line of slope 1, where actual LoS and predicted LoS are equal)

Figure 18 shows a density plot depicting the relationship between the predicted length of stay and the actual length of stay.

**Fig. 19:**
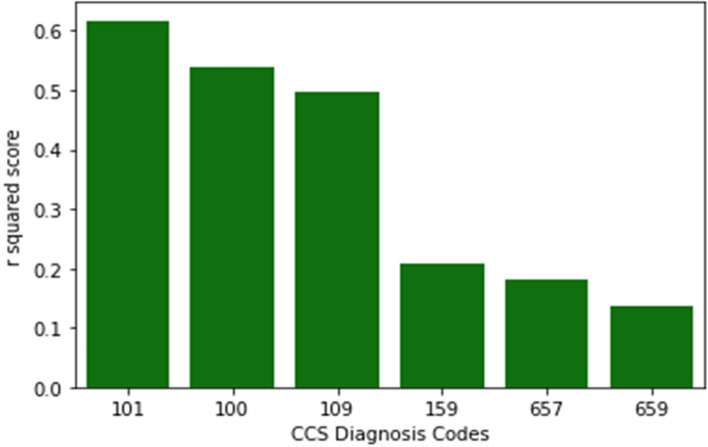
This figure shows the three CCS diagnosis codes that produced the top three R^2^ scores using linear regression. These are 101, 100 and 109. The three CCS Diagnosis codes that produced the lowest R^2^ scores are 159, 657, and 659

Most of the existing literature on LoS stay prediction is based on data for specific disease conditions such as cancer or cardiac disease. Hence, in order to understand which CCS diagnosis codes produce good model fits, we produced the plot in Fig. 19.

We provide the following descriptions in Tables 4 and 5 for the 3 CCS Diagnosis Codes in Fig. 19 with the top R^2^ Scores using linear regression.

**Table 4:** CCS Diagnosis codes, descriptions and R^2^ Scores for the top 3 CCS codes in Fig. 19

CCS Diagnosis Code	CCS Diagnosis Description	R^2^ Score
101	Coronary atherosclerosis and other heart disease	0.617
100	Acute myocardial infarction	0.538
109	Acute cerebrovascular disease	0.497

**Table 5:** CCS Diagnosis codes, descriptions and R^2^ Scores for the lowest 3 CCS codes in Fig. 19

CCS Diagnosis Code	CCS Diagnosis Description	R^2^ Score
159	Urinary tract infections	0.209
657	Mood disorders	0.182
659	Schizophrenia and other psychotic disorders	0.135

Similarly, the following table shows the 3 CCS Diagnosis Codes in Fig. 19 for the lowest R^2^ Scores using linear regression.

### Models with minimal feature sets

**Fig. 20:**
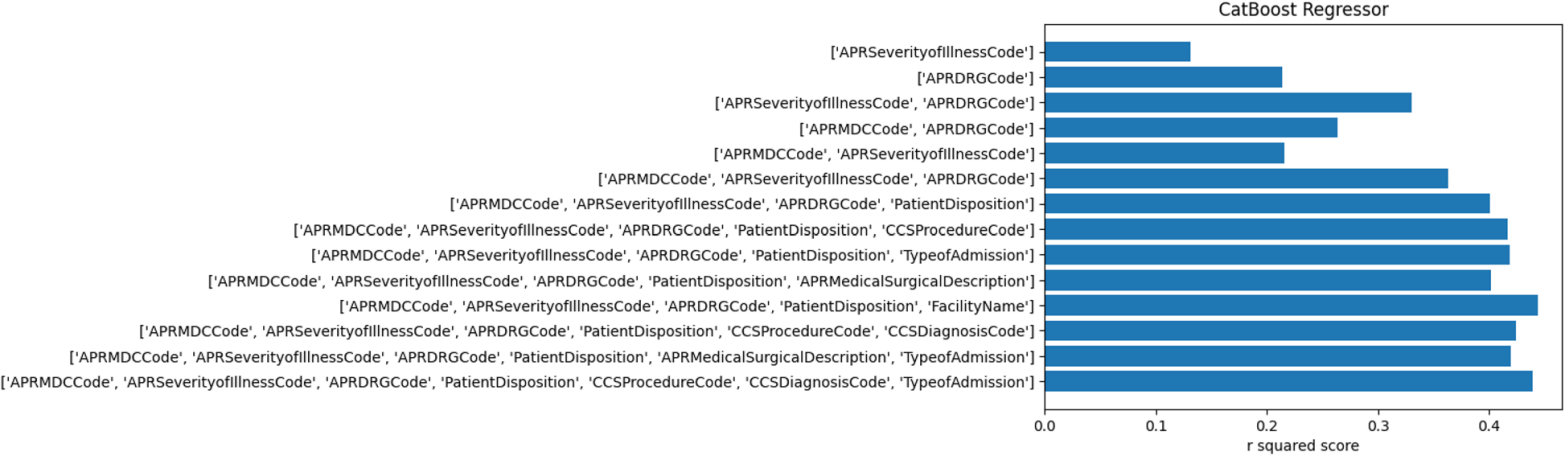
The labels for each row on the left show combinations of different input features. A CatBoost regression model was developed using the selected combination of features. The R^2^ correlation scores for each model is shown in the bar graph

We trained a CatBoost Regressor [65] on the complete dataset in order to determine the relationship between combinations of features and the prediction accuracy as determined by the R^2^ correlation score. This is shown in Fig. 20

We can infer from Fig. 20 that only four features (‘'APR MDC Code', 'APR Severity of Illness Code', 'APR DRG Code', 'Patient Disposition') are sufficient for the model to reach very close to its maximum performance. We obtain similar concurring results when using other regression models for the same experiment.

### Classification trees

**Fig. 21:**
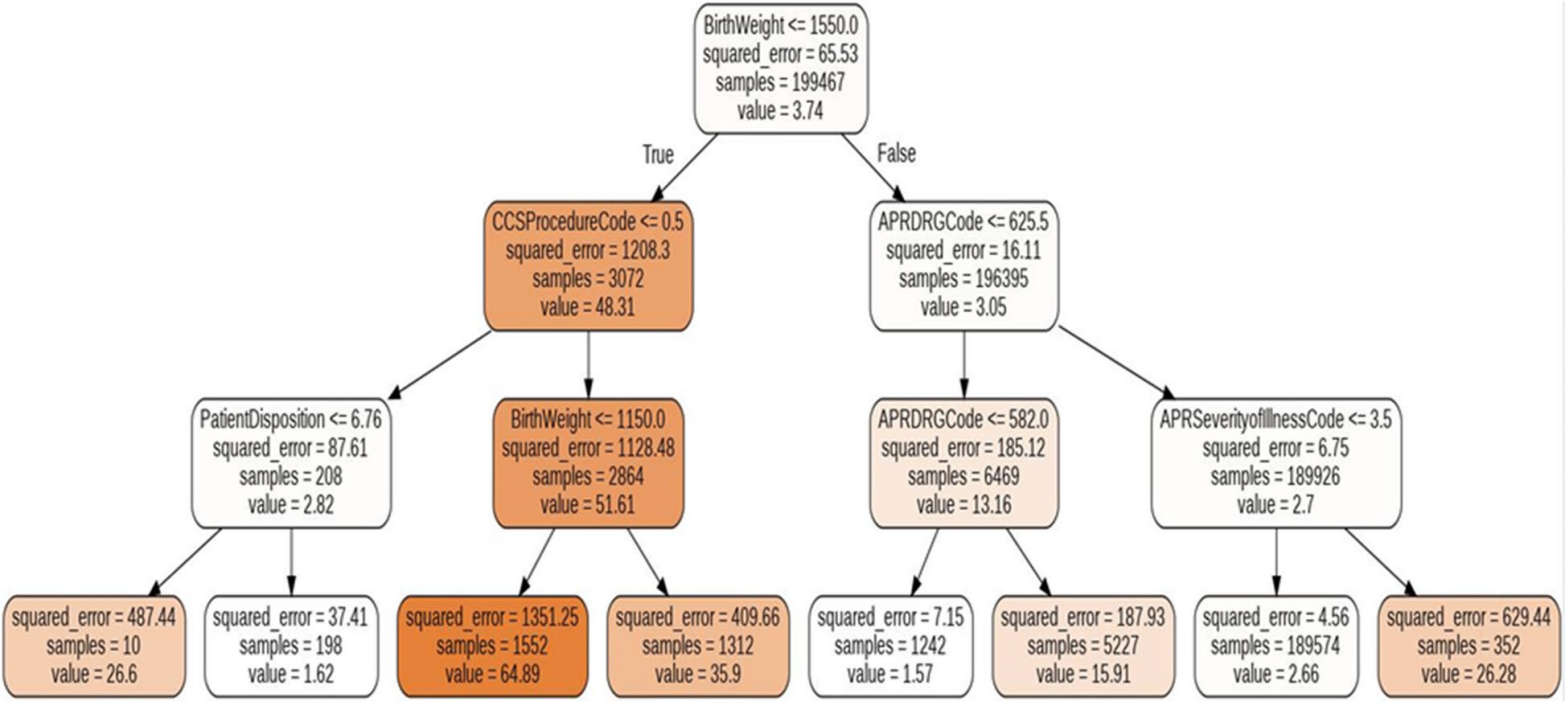
A random forest tree that represents a best-fit model to the data for newborns. With 4 levels of the decision tree, the R^2^ score is 0.65

**Fig. 22:**
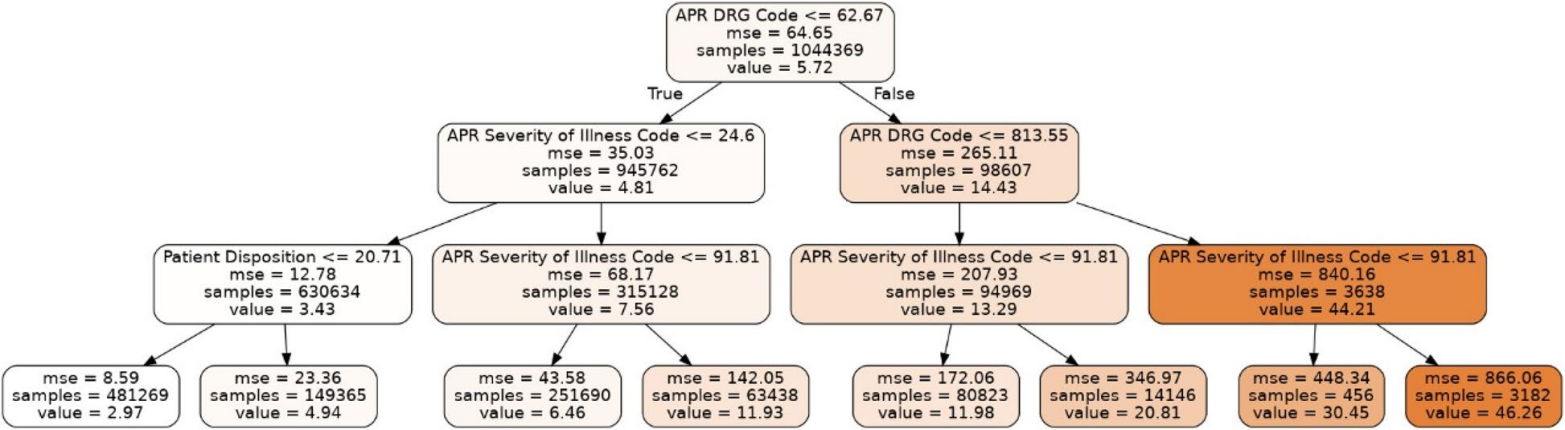
A random forest tree using only a tree of depth 3 that represents a best-fit model to the data for non-newborns. The R^2^ score is 0.28. We can generate trees with greater depth that better fit the data, but we have shown only a depth of 3 for the sake of readability in the printed version of this paper. Otherwise, the tree would be too large to be legible on this page. The main point in this figure is to showcase the ease of interpretation of the working of the model through rules

We used a random forest tree approach to generate the trees in Figs. 21 and 22.

### Model performance measures

#### Regression

We used tenfold cross validation to determine the regression scores. The results are summarized in Tables 6 and 7.

**Table 6:** This table summarizes the R^2^ scores for three different regression models we investigated. This computation is for non-newborns

Model name	R^2^ score	*p* value
Catboost regression	0.432	< 1 e -2
Random Forest Regression	0.396	< 1 e -2
Linear Regression	0.42	< 1 e -2

**Table 7:** This table summarizes the R^2^ scores for three different regression models we investigated. This computation is for newborns

Model name	R^2^ score	*p* value
Catboost regression	0.730	< 1 e -2
Random Forest Regression	0.767	< 1 e -2
Linear Regression	0.82	< 1 e -2

#### Classification

We computed the multi-class classifier metrics for logistic regression, using one-hot encoding for non-newborns. The results are presented in Table 8. The first row represents the accuracy of the classifier when Class 0 is compared against the rest of the classes. A similar interpretation applies to the other rows in the table, ie one-versus-rest. The macro average gives the balanced recall and precision, and the resulting F1 score. The weighted average gives a support (number of samples) weighted average of the individual class metric. The overall accuracy is computed by dividing the total number of accurate predictions, which is 49,686 out of a total number of 105,932 samples, which yields a value of 0.47.

**Table 8:** Evalution of multi-class classifier metrics for logistic regression for non-newborns. The macro-averaged scores are computed using the arithmetic mean of all the per-class scores. The weighted average scores are computed by using the support values as the weights

	Precision	Recall	F1-score	Support
Class 0	0.45	0.56	0.50	16,685
Class 1	0.44	0.40	0.42	21,235
Class 2	0.57	0.11	0.19	18,520
Class 3	0.38	0.49	0.43	25,161
Class 4	0.59	0.71	0.65	24,331
Macro avg	0.49	0.46	0.44	105,932
Weighted avg	0.48	0.47	0.45	105,932

**Fig. 23:**
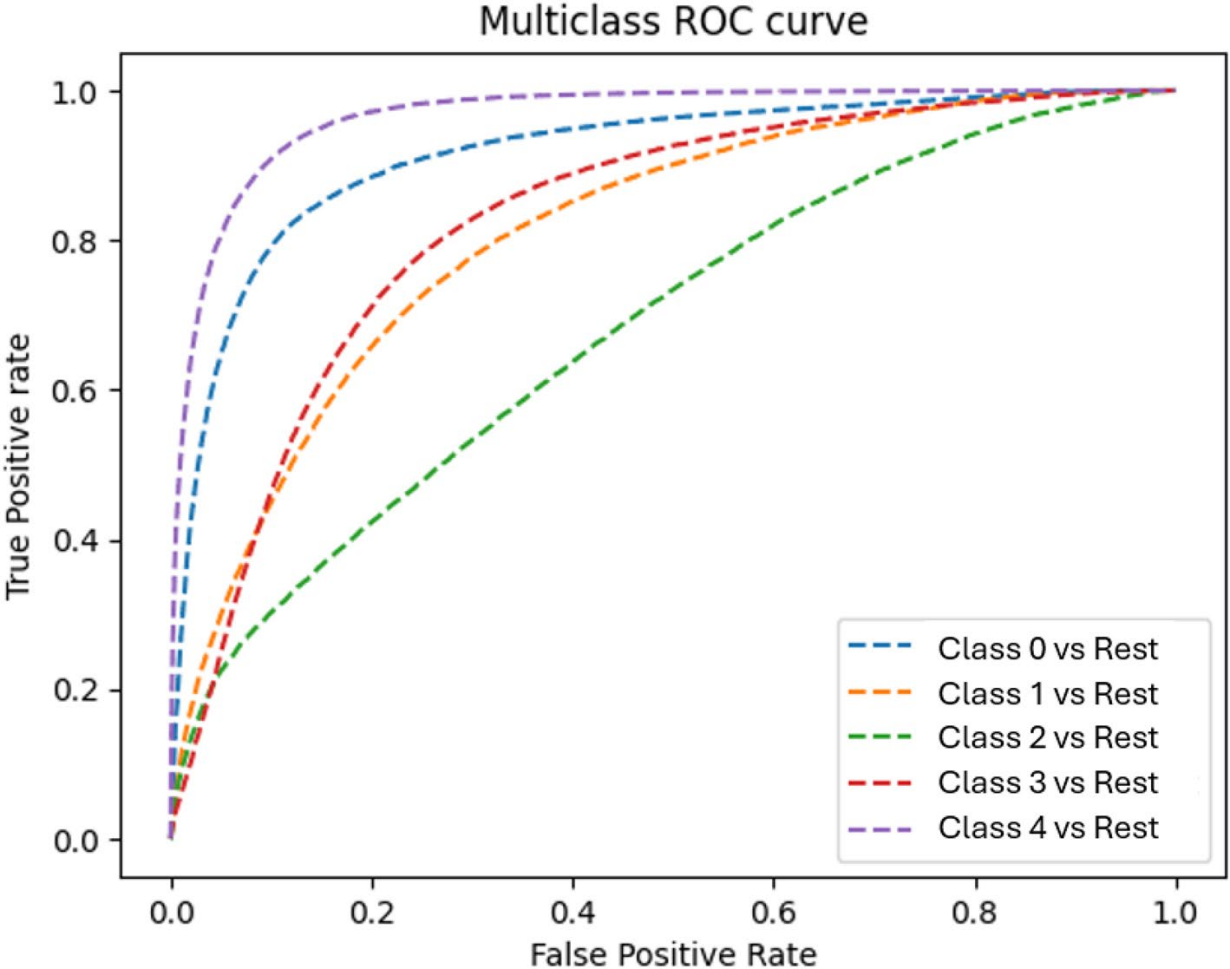
This figure applies to data concerning non-newborns. We show the multiclass ROC curves for the performance of the catboost classifier for the different classes shown. The area under the ROC curve is 0.7844

For the category of non-newborns, Fig. 23 provides a graphical plot that visualizes the ROC curves for the different multiclass classifiers we developed.

In Table 9 we compare the performance of our multiclass classifier using logistic regression developed on 2016 SPARCS data against 2017 SPARCS data.

**Table 9:** In the first scenario, we developed a multiclass classifier using logistic regression with the 2016 SPARCS dataset. The performance of the classifier is shown for the year 2016. In the second scenario, we used this trained classifier against the 2017 SPARCS dataset. This table compares the performance of the classifier for the categories of newborns and non-newborns in these two scenarios

Category: Newborns	Year	Accuracy
	2016	0.605
	2017	0.604
Category: Non-newborns	Year	Accuracy
	2016	0.606
	2017	0.590

**Fig. 24:**
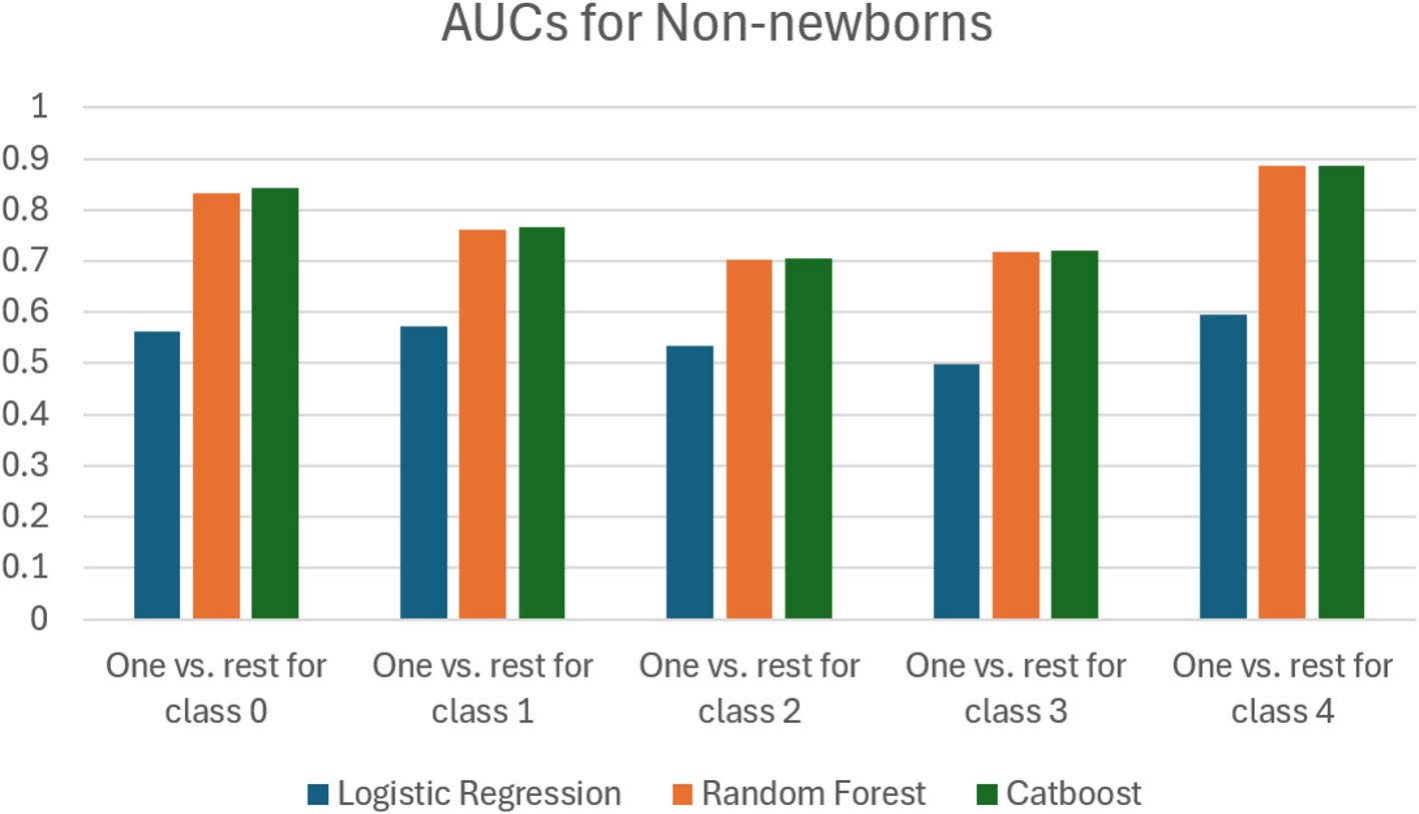
A bar chart that depicts the data in Table 10 for non-newborns

**Fig. 25:**
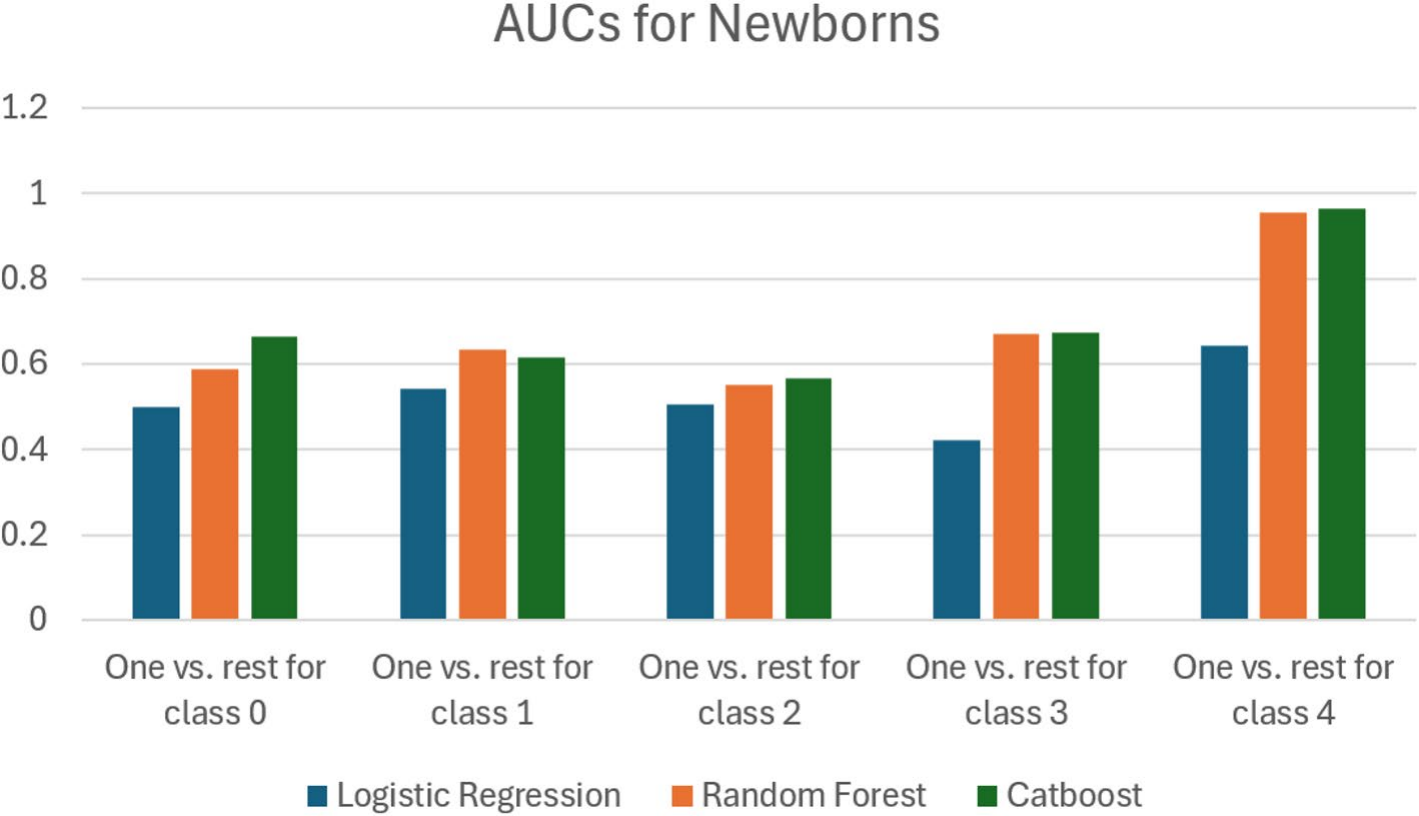
A bar chart that depicts the data in Table 11

In order to compare the performance of the different classifiers, we computed the AUC measures reported in Table 10. Figure 24 visualizes the data in Table 10 and Fig. 25 visualizes the data in Table 11. In Tables 12 and 13 we report the results of computing the Delong test for non-newborns and newborns respectively. In Tables 14 and 15 we report the results of computing the Brier scores for non-new borns and newborns respectively.

**Table 10:** We report the AUC scores for the three different classifiers we used, logistic regression, random forest and catboost. This is for the case of non-newborns. The last column computes the average AUC over the previous columns

	Binary classes used					Average AUC
Classifier used	One vs. rest for class 0	One vs. rest for class 1	One vs. rest for class 2	One vs. rest for class 3	One vs. rest for class 4	
Logistic Regression	0.561	0.573	0.534	0.498	0.595	0.5522
Random Forest	0.832	0.762	0.702	0.719	0.885	0.78
Catboost	0.842	0.767	0.705	0.721	0.887	0.7844

**Table 11:** We report the AUC scores for the three different classifiers we used, logistic regression, random forest and catboost. This is for the case of newborns. The last column computes the average AUC over the previous columns

	Binary classes used					Average AUC
Classifier used	One vs. rest for class 0	One vs. rest for class 1	One vs. rest for class 2	One vs. rest for class 3	One vs. rest for class 4	
Logistic Regression	0.498	0.541	0.504	0.424	0.643	0.522
Random Forest	0.589	0.635	0.550	0.671	0.954	0.6798
Catboost	0.664	0.615	0.565	0.673	0.964	0.6962

**Table 12:** This table uses data for non-newborns. We report the results of using the Delong test to conduct a pairwise comparison of the AUCs generated by two models at a time. For each model, measured the performance of binary classifiers, designated as “One vs. rest for Class 0”, “One vs. rest for Class 1” and so on. A positive value for the Delong test statistic indicates that the AUC for the first model is larger than the AUC for the second model

Binary classes used	Models compared	Delong test statistic	*p*-value
One vs. rest for Class 0	Logistic regression vs. Random Forests	-153.156	0.0
	Random Forests vs. Catboost	-29.575	0.0
	Catboost vs. Logistic regression	157.182	0.0
One vs. rest for Class 1	Logistic regression vs. Random Forests	-139.057	0.0
	Random Forests vs. Catboost	-16.858	0.0
	Catboost vs. Logistic regression	143.066	0.0
One vs. rest for Class 2	Logistic regression vs. Random Forests	-104.347	0.0
	Random Forests vs. Catboost	-8.677	< 1 e -10
	Catboost vs. Logistic regression	106.118	0.0
One vs. rest for Class 3	Logistic regression vs. Random Forests	-143.625	0.0
	Random Forests vs. Catboost	-2.68	0.004
	Catboost vs. Logistic regression	144.197	0.0
One vs. rest for Class 4	Logistic regression vs. Random Forests	-187.196	0.0
	Random Forests vs. Catboost	-10.278	< 1 e -10
	Catboost vs. Logistic regression	188.001	0.0

**Table 13:** This table uses data for newborns. We report the results of using the Delong test to conduct a pairwise comparison of the AUCs generated by two models at a time. For each model, we measured the performance of binary classifiers, designated as “One vs. rest for Class 0”, “One vs. rest for Class 1” and so on. A positive value for the Delong test statistic indicates that the AUC for the first model is larger than the AUC for the second model

Binary classes used	Models compared	Delong test statistic	*p*-value
One vs. rest for Class 0	Logistic regression vs. Random Forests	-11.83	< 1 e -10
	Random Forests vs. Catboost	-12.102	< 1 e -10
	Catboost vs. Logistic regression	21.475	< 1 e -10
One vs. rest for Class 1	Logistic regression vs. Random Forests	-24.305	< 1 e -10
	Random Forests vs. Catboost	6.823	< 1 e -10
	Catboost vs. Logistic regression	18.098	< 1 e -10
One vs. rest for Class 2	Logistic regression vs. Random Forests	-9.958	< 1 e -10
	Random Forests vs. Catboost	-5.077	1.920 e -07
	Catboost vs. Logistic regression	13.541	< 1 e -10
One vs. rest for Class 3	Logistic regression vs. Random Forests	-28.775	< 1 e -10
	Random Forests vs. Catboost	-0.914	0.180
	Catboost vs. Logistic regression	29.66	< 1 e -10
One vs. rest for Class 4	Logistic regression vs. Random Forests	-35.148	< 1 e -10
	Random Forests vs. Catboost	-8.06	< 1 e -10
	Catboost vs. Logistic regression	36.011	< 1 e -10

**Table 14:** We report the Brier scores computed for the performance of the different classifier models we developed. This table uses data from non-newborns

Type of classifier	Brier score
Logistic Regression	0.754
Random Forest classifier	0.644
Catboost classifier	0.635

**Table 15:** We report the Brier scores computed for the performance of the different classifier models we developed. This table uses data from newborns

Type of classifier	Brier score
Logistic Regression	0.780
Random Forest classifier	0.532
Catboost classifier	0.635

#### Model parameters

In Table 16 we present the parameter and hyperparameter values used in the different models.

**Table 16:** Model parameter and hyperparameter values used

Type of Model	Parameter	Value
Logistic Regression		
	Adam optimzer, Learning rate	1e-3
	Adam optimizer, Weight decay	1e-4
	Adam optimizer, Number of epochs	10
Random Forest Regression	Number of estimators	10
	Maximum depth	10
Decision Tree Regression	Maximum depth	5

### Additional results shown in the Appendix/Supplementary material

Due to space restrictions, we show additional results in the Appendix/Supplementary Material. These results are in tabular form and describe the R^2^ scores for different segmentations of the variables in the dataset, e.g. according to age group, severity of illness code, etc.

## Discussion

The most significant result we obtain is shown in Figs. 21 and 22, which provides an interpretable working of the decision trees using random forest modeling. Figure 21 for newborns shows that the birth weight features prominently in the decision tree, occurring at the root node. Low birth weights are represented on the left side of the tree and are typically associated with longer hospital stays. Higher birth weights occur on the right side of the tree, and the node in the bottom row with 189,574 samples shows that the most frequently occurring predicted stay is 2.66 days. Figure 22 for non-newborns shows that the features of “APR DRG Code”, “APR Severity of Illness Code” and “Patient Disposition” are the most important top-level features to predict the LoS. This provides a relatively simple rule-based model, which can be easily interpreted by healthcare providers as well as patients. For instance, the right-most branch of the tree classifies the input data into a relatively high LoS (46 days) when the branch conditions APR DRG Code is greater than 813.55 and the APR Severity of Illness Code is less than 91.

The results in Fig. 19 and Table 4 show that if we restrict our model to specific CCS Diagnosis descriptions such as “coronary atherosclerosis and other heart disease”, we obtain a good R^2^ Score of 0.62. The objective of our work is not to cherry-pick CCS Diagnosis codes that produce good results, but rather to develop a single model for the entire SPARCS dataset to obtain a birds-eye perspective. For future work, we can explicitly build separate models for each CCS Diagnosis code, and that could have relevance to specific medical specialties, such as cardiovascular care.

Similarly, the results in Fig. 19 and Table 5 show that there are CCS Diagnosis codes corresponding to schizophrenia and mood disorders that produce a poor model fit. Factors that contribute to this include the type of data in the SPARCS dataset, where information about patient vitals, medications, or a patient’s income level is not provided, and the inherent variability in treating schizophrenia and mood disorders. Baeza et al. [69] identified several variables that affect the LoS in psychiatric patients, which include psychiatric admissions in the previous years, psychiatric rating scale scores, history of attempted suicide, and not having sufficient income. Such variables are not provided in the SPARCS dataset. Hence a policy implication is to collect and make such data available, perhaps as a separate dataset focused on mental health issues, which have proven challenging to treat.

Figures 16 and 17 show that a better regression fit is obtained when a specific CCS Diagnosis code is used to build the model, such as “Newborn” in Fig. 16. To put these results in context, we note that it is difficult to obtain a high R^2^ value for healthcare datasets in general, and especially for large numbers of patient samples that span multiple hospitals. For instance, Bertsimas [70] reported an R^2^ value of 0.2 and Kshirsagar [71] reported an R^2^ value of 0.33 for similar types of prediction problems as studied in this paper.

Further details for a segmentation of R^2^ scores by the different variable categories are shown in the Appendix/Supplementary Material section. For instance, the table corresponding to Age Groups shows that there is close agreement between the mean of the predicted LoS from our model and the actual LoS. Furthermore, the mean LoS increases steadily from 4.8 days for Age group 0–17 to 6.4 days for ages 70 or older. A discussion of these tables is outside the scope of this paper. However, they are being provided to help other researchers form hypotheses for further investigations or to find supporting evidence for ongoing research.

Table 3 shows that the best encoding scheme is to combine target encoding with one-hot encoding and then apply linear regression. This produces an R^2^ score of 0.42 for the non-newborn data, which is the best fit we could obtain. This table also shows that significant improvements can be obtained by exploring the search space which consists of different strategies of feature encoding and regression methods. There is no theoretical framework which determines the optimum choice, and the best method is to conduct an experimental search. An important contribution of the current paper is to explore this search space so that other researchers can use and build upon our methodology.

The distribution of errors in Fig. 15 shows that the truncation we employed at a LoS of 8 days produces artifacts in the prediction model as all stays of greater than 8 days are lumped into one class. Nevertheless, the distribution of LoS values in Fig. 4 shows that a relatively small number of data samples have LoS greater than 8 days. In the future, we will investigate different truncation levels, and this is outside the scope of the current paper. By using our methodology, the truncation level can also be tuned by practitioners in the field, including hospital administrators and other researchers.

Our results in Fig. 7 show that certain features are not useful in predicting the LoS. The SHAP plot shows that features such as race, gender, and ethnicity are not useful in predicting the LoS. It would have been interesting if this were not the case, as that implies that there is systemic bias based on race, gender or ethnicity. For instance, a person with a given race may have a smaller LoS based on their demographic identity. This would be unacceptable in the medical field. It is satisfying to see that a large and detailed healthcare dataset does not show evidence of bias.

To place this finding in context, racial bias is an important area of research in the U.S., especially in fields such as criminology and access to financial services such as loans. In the U.S., it is well known that there is a disproportional imprisonment of black and Hispanic males [72]. Researchers working on criminal justice have determined that there is racial bias in the process of sentencing and granting parole, with blacks being adversely affected [73]. This bias is reinforced through any algorithms that are trained on the underlying data. There is evidence that banks discriminate against applicants for loans based on their race or gender [74].

This does not appear to be the case in our analysis of the SPARCS data. Though we did not specifically investigate the issue of racial bias in the LoS, the feature analysis we conducted automatically provides relevant answers. Other researchers including those in the U.K [21] have also determined that gender does not have an effect on LoS or costs. Hence the results in the current paper are consistent with the findings of other researchers in other countries working on entirely different datasets.

From Table 6 we see that in the case of data concerning non-newborns, the catboost regression performs the best, with an R^2^ score of 0.432. The *p*-value is less than 0.01, indicating that the correlation between the actual and predicted values of LoS through catboost regression is statistically significant. Similarly, the *p*-values for linear regression and random forest regression indicate that these models produce predictions that are statistically significant, i.e. they did not occur by random chance.

From Table 7 that refers to data from newborns, the linear regression performs the best, with an R^2^ score of 0.82. The *p*-value is less than 0.01, indicating that the correlation between the actual and predicted values of LoS through linear regression is statistically significant. Similarly, the *p*-values for random forest regression and catboost regression indicate that these models produce predictions that are statistically significant.

We examine the performance of classifiers on non-newborn data, as shown in Tables 10 and 12. The Delong test conducted in Table 12 shows that there is a statistically significant difference between the AUCs of the pairwise comparisons of the models. Hence, we conclude that the catboost classifier performs the best with an average AUC of 0.7844. We also note that there is a marginal improvement in performance when we use the catboost classifier instead of the random forest classifier. Both the catboost classifier and the random forest classifier perform better than logistic regression. We conclude that the best performing model for non-newborns is the catboost classifier, followed by the random forest classifier, and then logistic regression.

In the case of newborn data, we examine the performance of the classifiers as shown in Tables 11 and 13. From Table 13, we note that the *p*-values in all the rows are less than 0.05, except for the binary class “one vs. rest for class 3”, random forests vs. catboost. Hence, for this particular comparison between the random forest classifier and the catboost classifier for “one vs. rest for class 3”, we cannot conclude that there is a statistically significant difference between the performance of these two classifiers. From Table 11 we observe that the AUCs of these two classifiers are very similar. We also note that only about 10% of the dataset consists of newborn cases.

From Table 14 we note that the Brier score for the catboost classifier is the lowest. A lower Brier score indicates better performance. According to the Brier scores for the non-newborn data, the catboost classifier performs the best, followed by the random forest classifier and then logistic regression. Table 15 shows that for newborns, the random forest classifier performs the best, followed by the catboost classifier and logistic regression. The performance of the random forest classifier and catboost classifier are very similar.

From a practical perspective, it may make sense to use a catboost classifier on both newborn and non-newborn data as it simplifies the processing pipeline. The ultimate decision rests with the administrators and implementers of these decision systems in the hospital environment.

Burn et al. observe [21] that though the U.S. has reported similar declines in LoS as in the U.K, the overall costs of joint replacement have risen. The U.K. government created policies to encourage the formation of specialist centers for joint replacement, which have resulted in reduction in the LoS as well as delivering cost reductions. The results and analysis presented in our current paper can help educate patients and healthcare consumers about trends in healthcare costs and how they can be reduced. An informed and educated electorate can press their elected representatives to make changes to the healthcare system to benefit the populace.

Hachesu et al. examined the LoS for cardiac disease patients [22] where they used data from around 5000 patients and considered 35 input variables to build a predictive model. They found that the LoS was longer in patients with high blood pressure. In contrast, our method uses data from 2.5 million patients and considers multiple disease conditions simultaneously. We also do not have access to patient vitals such as blood pressure measurements, due to the limitation of the existing New York State SPARCS data.

Garcia et al. [23] conducted a study of elderly patients (age greater than 60) to understand factors governing the LoS for hip fracture treatment. They used 660 patient records and determined that the most significant variable was the American Society of Anesthesiologists (ASA) classification system. The ASA score ranges from 1–5 and captures the anesthesiologist’s impression of a patient’s health and comorbidities at the time of surgery. Garcia et al. showed a monotonically increasing relationship between the ASA score and the LoS. However, they did not build a specific predictive model. Their work shows that it is possible to find single variables with significant information content in order to estimate the LoS. The New York SPARCS dataset that we used does not contain the ASA score. Hence a policy implication of our research is to alert the healthcare authorities include such variables such as the ASA score where relevant in the datasets released in the future. The additional storage required is very small (one additional byte per patient record).

Arjannikov et al. [25] developed predictive models by binarizing the data into two categories, e.g. LoS <  = 2 days or LoS > 2 days. In our work, we did not employ such a discretization. In contrast, we used continuous regression techniques as well as classification into more than two bins. It is preferable to stay as close to the actual data as possible.

Almashrafi et al. [27] and Cots et al. [75] observed that larger hospitals tended to have longer LoS for patients undergoing cardiac surgery. Though we did not specifically examine cardiac surgery outcomes, our feature analysis indicated that the hospital operating certificate number had lower relevance than other features such as DRG codes. Nevertheless, the SHAP plots in Fig. 7 and Fig. 8 show that the hospital operating certificate number occurs within the top 10 features in order of SHAP values. We will investigate this relationship in more detail in future research, as it requires determining the size of the hospital from the operating certificate number and creating an appropriate machine-learning model. The Appendix contains results that show certain operating certificate numbers that produce a good model fit to the data.

A major focus of our research is on building interpretable and explainable models. Based on the principle of parsimony, it is preferable to utilize models which involve fewer features. This will provide simpler explanations to healthcare professionals as well as patients. We have shown through Fig. 20 that a model with five features performs just as well as a model with seven features. These features also make intuitive sense and the model’s operation can be understood by both patients and healthcare providers.

Patients in the U.S. increasingly have to pay for medical procedures out-of-pocket as insurance payments do not cover all the expenses, leading to unexpectedly large bills [76]. Many patients also do not possess health insurance in the U.S., with the consequence that they get charged the highest [77]. Kullgreen et.al. observe that patients in the U.S. need to be discerning healthcare consumers [78], as they can optimize the value they receive from out-of-pocket spending. In addition to estimating the cost of medical procedures, patients will also benefit from estimating the expected duration for a procedure such as joint replacement. This will allow them to budget adequate time for their medical procedures. Patients and consumers will benefit from obtaining estimates from an unbiased open data source such as New York State SPARCS and the use of our model.

Other researchers have developed specific LoS models for particular health conditions, such as cardiac disease [22], hip replacement [21], cancer [26], or COVID-19 [24]. In addition, researchers typically assume a prior statistical distribution for the outcomes, such a Weibull distribution [24]. However, we have not made any assumptions of specific prior statistical distributions, nor have we restricted our analysis to specific diseases. Consequently, our model and techniques should be more widely applicable, especially in the face of rapidly changing disease trajectories worldwide.

Our study is based exclusively on freely available open health data. Consequently, we cannot control the granularity of the data and must use the data as-is. We are unable to obtain more detailed patient information such as their physiological variables such as blood pressure, heartrate variability etc. at the time of admittance and during their stay. Hospitals, healthcare providers, and insurers have access to this data. However, there is no mandate for them to make this available to researchers outside their own organizations. Sometimes they sell de-identified data to interested parties such as pharmaceutical companies [79]. Due to the high costs involved in purchasing this data, researchers worldwide, especially in developing countries are at a disadvantage in developing AI algorithms for healthcare.

There is growing recognition that medical researchers need to standardize data formats and tools used for their analysis, and share them openly. One such effort is the organization for Observational Health Data Sciences and Informatics (OHDSI) as described in [80].

Twitter has demonstrated an interesting path forward, where a small percentage of its data was made available freely to all users for non-commercial purposes through an API [81]. Recently, Twitter has made a larger proportion of its data available to qualified academic researchers [82]. In the future, the profit motives of companies need to be balanced with considerations for the greater public good. An advantage of using the Twitter model is that it spurs more academic research and allows universities to train students and the workforce of the future on real-world and relevant datasets.

In the U.S., a new law went into effect in January 2021 requiring hospitals to make pricing data available publicly. The premise is that having this data would provide better transparency into the working of the healthcare system in the U.S. and lead to cost efficiencies. However, most hospitals are not in compliance with this law [83]. Concerted efforts by government officials as well as pressure by the public will be necessary to achieve compliance. If the eventual release of such data is not accompanied by a corresponding interest shown by academicians, healthcare researchers, policymakers, and the public it is likely that the very premise of the utility of this data will be called into question. Furthermore, merely dumping large quantities of data into the public domain is unlikely to benefit anyone. Hence research efforts such as the one presented in this paper will be valuable in demonstrating the utility of this data to all stakeholders.

Our machine-learning pipeline can easily be applied to new data that will be released periodically by New York SPARCS, and also to hospital pricing data [83]. Due to our open-source methodology, other researchers can easily extend our work and apply it to extract meaning from open health data. This improves reproducibility, which is an essential aspect of science. We will make our code available on Github to interested researchers for non-commercial purposes.

### Limitations of our models

Our models are restricted to the data available through New York State SPARCS, which does not provide detailed information about patient vitals. More detailed physiological data is available through the Multiparameter Intelligent Monitoring in Intensive Care (MIMIC) framework [84], though for a smaller number of patients. We plan to extend our methodology to handle such data in the future. Another limitation of our study is that it does not account for patient co-morbidities. This arises from the de-identification process used to release the SPARCS data, where patient information is removed. Hence we are unable to analyze multiple hospital admissions for a given patient, possibly for different conditions. The main advantage of our approach is that it uses large-scale population data (2.3 million patients) but at a coarse level of granularity, where physiological data is not available. Nevertheless, our approach provides a high-level view of the operation of the healthcare system, which provides valuable insights.

## Conclusion

There is growing interest in using data analytics to increase government transparency and inform policymaking. It is expected that the meaning and insights gained from such evidence-based analysis will translate to better policies and optimal usage of the available infrastructure. This requires cooperation between computer scientists, domain experts, and policy makers. Open healthcare data is especially valuable in this context due to its economic significance. This paper presents an open-source analytics system to conduct evidence-based analysis on openly available healthcare data.

The goal is to develop interpretable machine learning models that identify key drivers and make accurate predictions related to healthcare costs and utilization. Such models can provide actionable insights to guide healthcare administrators and policy makers. A specific illustration is provided via a robust machine learning pipeline that predicts hospital length of stay across 285 disease categories based on 2.3 million de-identified patient records. The length of stay is directly related to costs.

We focused on the interpretability and explainability of input features and the resulting models. Hence, we developed separate models for newborns and non-newborns, given differences in input features. The best performing model for non-newborn data was catboost regression, which used linear regression and achieved an R^2^ score of 0.43. The best performing model for newborns and non-newborns respectively was linear regression, which achieved an R^2^ score of 0.82. Key newborn predictors included birth weight, while non-newborn models relied heavily on the diagnostic related group classification. This demonstrates model interpretability, which is important for adoption. There is an opportunity to further improve performance for specific diseases. If we restrict our analysis to cardiovascular disease, we obtain an improved R^2^ score of 0.62.

The presented approach has several desirable qualities. Firstly, transparency and reproducibility are enabled through the open-source methodology. Secondly, the model generalizability facilitates insights across numerous disease states. Thirdly, the technical framework can easily integrate new data while allowing modular extensions by the research community. Lastly, the evidence generated can readily inform multiple key stakeholders including healthcare administrators planning capacity, policy makers optimizing delivery, and patients making medical decisions.

### Supplementary Information

Supplementary Material 1.

## Data Availability

Data is publicly available at the website mentioned in the paper, https://www.health.ny.gov/statistics/sparcs/ There is an “About Us” tab in the website which contains all the contact details. The authors have nothing to do with this website as it is maintained by New York State.
