# Supplementary material for: Predicting hospital length of stay using machine learning on a large open health dataset
Source: BMC Health Serv Res. 2024 Jul 29;24:860. doi: 10.1186/s12913-024-11238-y (PMC11288104; doi:10.1186/s12913-024-11238-y)
Supplement: Supplementary file 1 — Supplementary Material 1. [file 12913_2024_11238_MOESM1_ESM.pdf]

Appendix/Supplementary material: R^2 Scores for Different categories :

In this Appendix, we present the R^2 scores for different segmentations of the dataset. This allows us to determine which variables in the data produce good fits as well as poor fits.

1. Age Group :

This table shows that there is close agreement between the mean of the predicted LOS from our model and the actual LOS. Furthermore, the mean LOS increases steadily from 4.8 days for Age group 0-17 to 6.4 days for ages 70 or older.

Age Group	R^2 Score	Ratio of Samples	Mean of Actual LOS	Mean of predicted LOS	Standard Deviation of LOS
0-17	0.45	0.05	4.80	5	8.93
50-69	0.43	0.32	6.20	6.23	8.67
30-49	0.41	0.20	4.99	5.10	7.72
70 or older	0.40	0.32	6.40	6.44	7.54
30-49	0.35	0.10	4.58	4.70	7.36

†: All Authors contributed equally to this manuscript
*: Corresponding author

2. APR DRG Description :

(a) Top 3 :

This table shows the top three R2 scores based on APR DRG Descriptions. There is a close correspondence between the mean and predicted values of the LOS.

APR DRG Description	R2 Score	Ratio of Samples	Mean of actual LOS	Mean of predicted LOS	Standard Deviation
Percutaneous coronary intervention w/o AMI	0.43	0.02	3.50	3.76	4.48
Hip joint replacement	0.40	0.01	3.94	4.06	3.96
CVA & precerebral occlusion w infarct	0.36	0.01	5.48	5.56	5.63

(b) Bottom 3 :

APR DRG Description	R2 Score	Ratio of Samples	Mean of actual LOS	Mean of predicted LOS	Standard Deviation
Schizophrenia	0.12	0.01	18.97	19.15	20.75
Cesarean delivery	0.08	0.03	4.12	4.24	3.63
Vaginal delivery	0.05	0.05	2.55	2.53	1.67

3. APR MDC Code :

(a) Top 3 :

APR MDC Code	R2 Score	Ratio of Samples	Mean of actual LOS	Mean of predicted LOS	Standard Deviation
Diseases and Disorders of the Circulatory System	0.48	0.15	5.08	5.22	6.72
Diseases and Disorders of the Male Reproductive System	0.47	0.01	3.71	3.79	5.86
Diseases and Disorders of the Digestive System	0.46	0.1	5.17	5.3	6.56

(b) Bottom 3 :

APR MDC Code	R2 Score	Ratio of Samples	Mean of actual LOS	Mean of predicted LOS	Standard Deviation
Diseases and Disorders of the Skin, Subcutaneous Tissue and Breast	0.31	0.03	4.72	4.77	6.27
Mental Diseases and Disorders	0.2	0.05	12.11	12.28	15.22
Pregnancy, Childbirth and the Puerperium	0.15	0.09	3.08	3.1	2.96

4. APR Severity of Illness Description :

APR Severity of Illness Description	R2 Score	Ratio of Samples	Mean of actual LOS	Mean of predicted LOS	Standard Deviation
Extreme	0.35	0.08	14.88	14.83	16.04
Minor	0.33	0.26	3.29	3.36	4.3
Moderate	0.32	0.4	4.67	4.72	6.26
Major	0.28	0.26	7.09	7.21	7.67

5. APR Medical Surgical Description :

APR Medical Surgical Description	R2 Score	Ratio of Samples	Mean of actual LOS	Mean of predicted LOS	Standard Deviation
Surgical	0.53	0.28	6.61	6.75	9.61
Medical	0.34	0.72	5.49	5.53	7.33

6. APR Risk of Mortality :

APR Risk of Mortality	R2 Score	Ratio of Samples	Mean of actual LOS	Mean of predicted LOS	Standard Deviation
Extreme	0.39	0.07	13.94	13.75	15.27
Minor	0.36	0.51	4.15	4.2	6.1
Moderate	0.33	0.25	5.42	5.49	6.43
Major	0.31	0.18	7.84	8.05	8.92

7. CCS Diagnosis Description :

(a) Top 3 :

CCS Diagnosis Description	R2 Score	Ratio of Samples	Mean of actual LOS	Mean of predicted LOS	Standard Deviation
Coronary atherosclerosis and other heart disease	0.62	0.02	4.07	4.25	4.98
Acute myocardial infarction	0.54	0.02	5.26	5.29	6.79
Acute cerebrovascular disease	0.5	0.02	7.39	7.27	9.12

(b) Bottom 3 :

CCS Diagnosis Description	R2 Score	Ratio of Samples	Mean of actual LOS	Mean of predicted LOS	Standard Deviation
Urinary tract infections	0.21	0.02	4.89	4.91	5.34
Mood disorders	0.18	0.02	10.39	10.65	11.54
Schizophrenia and other psychotic disorders	0.14	0.02	17.45	17.74	19.8

8. CCS Procedure Description :

(a) Top 3 :

CCS Procedure Description	R2 Score	Ratio of Samples	Mean of actual LOS	Mean of predicted LOS	Standard Deviation
SPINAL FUSION	0.54	0.01	4.41	4.43	5.52
OT OR PRCS VES NOT HEAD	0.48	0.01	7.69	8	9.95
DX CARDIAC CATHETERIZTN	0.45	0.02	4.39	4.61	5.27

(b) Bottom 3 :

CCS Procedure Code	R2 Score	Ratio of Samples	Mean of actual LOS	Mean of predicted LOS	Standard Deviation
ARTHROPLASTY KNEE	0.15	0.02	3.32	3.22	2.38
CESAREAN SECTION	0.09	0.02	4.1	4.24	3.55
OT PRCS TO ASSIST DELIV	0.08	0.03	2.57	2.54	1.85

9. Emergency Department Indicator :

Emergency Department Indicator	R2 Score	Ratio of Samples	Mean of actual LOS	Mean of predicted LOS	Standard Deviation
N	0.45	0.33	5.64	5.73	8.62
Y	0.4	0.67	5.88	5.94	7.75

10. Facility Name :

(a) Top 5 :

Facility Name	R2 Score	Ratio of Samples	Mean of actual LOS	Mean of predicted LOS	Standard Deviation
Buffalo General Medical Center	0.56	0.01	6.02	6.14	8.34
Montefiore Med Center - Jack D Weiler Hosp of A Einstein College Div	0.55	0.01	5.28	5.32	7.05
New York Methodist Hospital	0.5	0.01	5.71	5.9	6.07
Winthrop-University Hospital	0.5	0.02	5.27	5.51	6.09
Albany Medical Center Hospital	0.5	0.02	6.07	6.2	7.64

(b) Last 4 :

Facility Name	R2 Score	Ratio of Samples	Mean of actual LOS	Mean of predicted LOS	Standard Deviation
Long Island Jewish Medical Center	0.37	0.02	6.05	6.02	8.65
Mount Sinai Beth Israel	0.36	0.01	5.56	5.68	6.77
University Hospital	0.36	0.01	5.89	5.86	8.72
NYU Hospitals Center	0.33	0.01	5.82	5.56	6.65

11. Type of Admission :

Type of Admission	R2 Score	Ratio of Samples	Mean of actual LOS	Mean of predicted LOS	Standard Deviation
Trauma	0.48	0	6.38	6.6	9.38
Elective	0.47	0.19	4.95	5.05	7.48
Urgent	0.45	0.08	6.6	6.62	9.58
Emergency	0.4	0.73	5.93	5.99	7.99

12. Operating Certificate Number :

(a) Top 3 :

Operating Certificate Number	R2 Score	Ratio of Samples	Mean of actual LOS	Mean of predicted LOS	Standard Deviation
2908000	0.5	0.02	5.27	5.51	6.09
101000	0.5	0.02	6.07	6.2	7.64
7001021	0.49	0.01	5.69	5.94	6.17

(b) Bottom 3 :

Operating Certificate Number	R2 Score	Ratio of Samples	Mean of actual LOS	Mean of predicted LOS	Standard Deviation
3301007	0.37	0.02	5.97	6.08	8.24
7002053	0.36	0.02	5.79	5.59	6.95
5151001	0.36	0.01	5.89	5.86	8.72
